# IL-33/ST2 induces macrophage-dependent ROS production and TRPA1 activation that mediate pain-like responses by skin incision in mice

**DOI:** 10.7150/thno.97856

**Published:** 2024-08-19

**Authors:** Ruoyao Xu, Yushuang Pan, Kaige Zheng, Muyan Chen, Chengyu Yin, Qimiao Hu, Jie Wang, Qing Yu, Peiyi Li, Yan Tai, Junfan Fang, Boyu Liu, Jianqiao Fang, Guihua Tian, Boyi Liu

**Affiliations:** 1 Department of Neurobiology and Acupuncture Research, The Third Clinical Medical College, Key Laboratory of Acupuncture and Neurology of Zhejiang Province, Zhejiang Chinese Medical University, Hangzhou, China.; 2 Department of Rehabilitation in Traditional Chinese Medicine, the Second Affiliated Hospital of Zhejiang University School of Medicine, Hangzhou, China.; 3 Academy of Chinese Medical Sciences, Zhejiang Chinese Medical University, Hangzhou, China.; 4 Dongzhimen Hospital, Beijing University of Chinese Medicine, Beijing, China.

**Keywords:** Incisional pain, IL-33, macrophage, ROS, TRA1, ST2

## Abstract

**Background:** Insufficiently managed incisional (INC) pain severely affects patients' life quality and rehabilitation after a major operation. However, mechanisms underlying INC pain still remain poorly understood.

**Methods:** A mouse model of INC pain was established by skin plus deep muscle incision. Biochemistry assay, *in vivo* reactive oxygen species (ROS) imaging, Ca^2+^ imaging combined with retrograde labelling, neuron tracing and nocifensive behavior test, etc. were utilized for mechanism investigation.

**Results:** We found pro-nociceptive cytokine interleukin -33 (IL-33) ranked among top up-regulated cytokines in incised tissues of INC pain model mice. IL-33 was predominantly expressed in keratinocytes around the incisional area. Neutralization of IL-33 or its receptor suppression of tumorigenicity 2 protein (ST2) or genetic deletion of *St2* gene (*St2*^-/-^) remarkably ameliorated mechanical allodynia and improved gait impairments of model mice. IL-33 contributes to INC pain by recruiting macrophages, which subsequently release ROS in incised tissues via ST2-dependent mechanism. Transfer of excessive macrophages enhanced oxidative injury and reproduced mechanical allodynia in *St2*^-/-^ mice upon tissue incision. Overproduced ROS subsequently activated functionally up-regulated transient receptor potential ankyrin subtype-1 (TRPA1) channel innervating the incisional site to produce mechanical allodynia. Neither deleting *St2* nor attenuating ROS affected wound healing of model mice.

**Conclusions:** Our work uncovered a previously unrecognized contribution of IL-33/ST2 signaling in mediating mechanical allodynia and gait impairment of a mouse model of INC pain. Targeting IL-33/ST2 signaling could be a novel therapeutic approach for INC pain management.

## Introduction

Postoperative pain is termed as a pain condition present in patients following the surgery 1. If insufficiently managed, it can result in extended stays in hospital, complications and poor recoveries among the patients 1, 2. Postoperative pain still poses a major clinical challenge for the anesthesiologists and pain doctors 1-4. Currently, pain control approaches for postoperative pain usually rely on opioids and nonsteroidal anti-inflammatory drugs (NSAIDs) 5, 6. Frequent usage of these medications can cause a lot of adverse effects, including tolerance, addiction, peptic ulcers and renal dysfunction, etc. 7. Constant usage of opioids may further promote chronic pain since these drugs can worsen hyperalgesia 7, 8. Thus, it is important to investigate the mechanisms underlying postoperative pain, which may further help to identify novel targets for postoperative pain control.

One of the prominent reasons for postoperative pain etiology is tissue inflammation 9. After the surgery, a plethora of immune cells, including neutrophils, monocytes and macrophages, infiltrate to the injured site 10. These immune cells can release a panel of pro-inflammatory cytokines and chemokines that initiate complex and dynamic interactions with the nervous system via neuro-immune crosstalk 11, 12. After skin damage, keratinocytes also release a variety of inflammatory mediators, including adenosine triphosphate, nerve growth factor, interleukin-33 (IL-33), and other mediators that can act upon receptors expressed on sensory neurons 13. These inflammatory mediators or cytokines can directly activate sensory neurons to produce pain 14. Additionally, they may also sensitize sensory neurons and further exacerbate pain via peripheral sensitization 15. However, the exact mechanisms underlying the neuro-immune crosstalk during postoperative pain are still not completely understood.

IL-33 belongs to IL-1 family and acts via IL-33 specific ST2 and IL-1 receptor accessory protein (IL-1RAcp) receptor complex to contribute to inflammation, autoimmune and homeostasis process 16, 17. Recent work indicates IL-33 can serve as a pivotal pain mediator for a variety of pain conditions, e.g. bone cancer pain, muscle pain and neuropathic pain, etc. 18-20. Our group showed that IL-33 is released from keratinocytes upon skin inflammation and acts upon ST2 expressed in sensory neurons to drive sensory neuron hyperexcitability 21-23. Our work further showed that IL-33 released by macrophages promotes neutrophil-dependent ROS production via ST2 in the inflamed joint, contributing to gouty arthritis pain 24. These work in all suggests that IL-33 can serve as an important mediator for neuro-immune crosstalk that contributes to pain mechanisms via activating ST2 receptor expressed on inflammatory cells or sensory neurons 18.

In this study, our intention is to explore the potential pro-nociceptive or pro-inflammatory cytokines in the incised skin of a mouse model of incisional (INC) pain. We found that IL-33 was significantly up-regulated in keratinocytes of incised skin. IL-33 contributes to INC pain by recruiting macrophages influx and subsequent ROS production via ST2. ROS further activate functionally up-regulated TRPA1 channel that innervates the incised tissues of INC pain model mice and thereby mediate pain.

## Methods

### Experimental design

The aim of this work is to explore whether and how IL-33/ST2 signaling contributes to INC pain. To address this issue, we examined the distribution of IL-33 in incisional skin tissues and evaluated if blocking IL-33/ST2 signaling affects pain response of INC model mice. IL-33 contributes to INC pain by recruiting macrophages, which subsequently release ROS in incised tissues via ST2-dependent mechanism. We then performed adoptive transfer experiment and found transfer of excessive wildtype macrophages enhanced oxidative injury and reproduced mechanical allodynia in *St2*^-/-^ mice upon tissue incision. Then we studied whether TRPA1 channel innervating the incisional site was functionally up-regulated to contribute to peripheral sensitization. With the aid of the retrograde tracer WGA, we were able to find that ROS activated functionally up-regulated TRPA1 channel innervating the incisional site, resulting in INC pain. Lastly, we also assessed whether deleting ST2 or attenuating ROS might affect wound healing of INC model mice.

### Animals

Male BALB/c mice (6-8 weeks; 18-25 g) were purchased from Shanghai Laboratory Animal Center, Chinese Academy of Sciences. *St2* knockout (*St2*^-/-^) mice in the background of BALB/c were kindly provided by Dr. Andrew McKenzie at the MRC Laboratory of Molecular Biology, Cambridge, UK. All animals were housed in the Laboratory Animal Center of Zhejiang Chinese Medical University accredited by the Association for Assessment and Accreditation of Laboratory Animal Care (AAALAC) under standard environmental conditions (12 h light-dark cycle and 24°C). Food and water were provided *ad libitum*. The mice were randomly allocated and 5 mice were housed per cage. In this study, we only included male mice to circumvent possible hormonal female cycle interferences in the behavioral analysis.

### Skin plus deep tissue incisional pain model establishment

Anesthesia was induced in mice with 5% isoflurane in an induction chamber and maintained with 1.5% isoflurane using a nose cone. The plantar area of hind paw was disinfected using 70% alcohol before incision. A 5 mm long incision (2 mm away from the edge of the heel) was cut through the glabrous skin using a sterile scalpel blade. To further study deep tissue incision-induced pain, the underlying muscle was elevated with a sterile forceps and incised longitudinally, leaving muscle origin and insertion intact, as previously described 25. After the operation, the skin was sealed with sutures. The mice then underwent recover from anesthesia on a heating pad. Sutures were removed 1 day after the incision. The control mice (sham operation) receive the same anesthesia and disinfection as above but without incision.

### Mechanical allodynia

This method was used to measure mechanical hypersensitivity of mice. Mice were individually placed in transparent Plexiglas chambers on an elevated mesh floor and were habituated for 30 min before testing. The mechanical allodynia was determined using a series of von Frey filaments (from 0.04 to 4 g) applied perpendicularly to the plantar surface of the hind paw in ascending order beginning with the finest fiber. The minimum force that caused the mouse to withdraw its hind paw away from the filament was considered as the withdrawal threshold. For each mouse, a von Frey hair was applied 5 times at 10 s intervals. The threshold was determined when paw withdrawal was observed in more than 3 of 5 applications as we previously described 26, 27.

### Gait recording and analysis

This method was used to measure gait behavior affected by INC pain in mice. The gaits of mice was recorded and analyzed via DigiGait imaging system (MouseSpecifics, Inc., USA) as reported before 28, 29. The mouse was put on a flat and transparent treadmill, which operated on a constant speed (18 cm/s). A video camera was positioned underneath the apparatus to record gait of mice while running, and captured images of the illuminated area of each paw. The animals were allowed to run on the treadmill for 20 s and a consecutive 5 strides were averaged per animal and used for analysis. Parameters including paw area, stance, stride length and frequency were calculated via the software.

### Open field test

This method was used to evaluate locomotor activity of mice. The test consists of a non-transparent Plexiglas enclosure (size: 40 cm × 40 cm × 30 cm) placed in a sound-proof experimental room. Animals were habituated to experimental room conditions for 30 min before test. To initiate testing, the mice were placed in the center of the field individually, and the total distance they traveled in the field was monitored by a camera above the field during 5 min testing time. The movement was then analyzed by Anymaze software (Stoelting Co., USA). The box was wiped clean with 75% alcohol after each test. All behavior tests are conducted by an experimenter blinded to experimental conditions.

### Drug administration

IL-33 neutralizing antibody (2 μg/ mouse, #AF3626, R&D Systems, USA) or ST2 neutralizing antibody (6 μg/ mouse, #AF1004, R&D Systems, USA) was administered by intraplantar (i.pl.) injection at -1, 5, 23, 47 and 71 h time points, for a total of 5 times, after incision. Normal goat isotype control IgG were dissolved in sterile PBS and used as vehicle. Acetyl cysteine (NAC, an antioxidant, Beyotime Biotechnology, China), 2,2,6,6-tetramethylpiperidine-1-oxyl (Tempol, an antioxidant, Beyotime Biotechnology, China) was diluted in PBS and daily administered through intraperitoneal injection (200 mg/kg, i.p.) 1 h ahead of behavior test. Recombinant mouse IL-33 protein (R&D Systems, USA) was dissolved in 0.1% BSA (in PBS) and injected (intraplantar, 300 ng/site) into the incised skin tissues starting from Day 4 on a daily basis 1 h before behavioral test until Day 7. Vehicle group received 0.1% BSA in PBS injection. The CCR2 antagonist INCB3344 (APExBIO, USA) was dissolved in dimethyl sulfoxide (DMSO) as stock solution and further diluted to PBS. INCB3344 (10 μg/mouse/injection in 100 µl) was administered intravenously into the tail vein 2 h before INC pain model establishment and subsequently every day until Day 5. Vehicle group received 0.3% DMSO in PBS only. The dosage of INCB3344 was adopted as documented before 30.

### Extracellular IL-33 release assay

The mouse was euthanized beforehand and the ear was disinfected by 70% alcohol, cut and then incubated in DMEM (Thermo, USA) containing 10% fetal bovine serum (FBS, Hyclone, USA) and 100 U/mL penicillin and 100 μg/mL streptomycin in a 12-well plate at 24 °C for 3 h. Then the ear was washed 3 times with sterilized PBS and re-incubated in fresh culture medium to remove any potential IL-33 contamination due to ear excision. Three incisions (5 mm in length &2 mm apart) were then made by a sterilized blade in the ear skin of the incised group, whereas control group did not receive incision. After that, the ears were incubated for another 12 h. The culture medium was collected and centrifuged to remove any debris. Then the supernatant was collected and subject to ELISA assay for IL-33 release using a commercial ELISA kit (R&D Systems, USA).

### Retrograde labeling of DRG neurons

This method was used to label the DRG neurons that specifically innervate the incised area. The retrograde tracer iFluor 594-conjugated wheat germ agglutinin (WGA-594, 0.8%, AAT Bioquest, USA) was injected into the right foot pad 3 days before incision/sham operation was made. Injections were performed at 5 distinct sites (1 μl/site) around the area that was about to be incised. 3 days after INC/sham operation, mice were sacrificed and ipsilateral L3-L5 DRG neurons were isolated for further analysis.

### DRG neuron culture and Ca^2+^ imaging with WGA-labelled neurons

This method was used to monitor intracellular Ca^2+^ changes in DRG neurons, which is a reflection of neuron excitability. Ipsilateral L3-L5 dorsal root ganglion (DRG) innervating the hindpaw were dissociated 3 d after postoperative pain model establishment. DRG neurons were cultured in DMEM+10% FBS (Hyclone, USA) on round coverslips coated with poly-D-lysine (Sigma, USA) as previously described 31, 32. The DRG neurons were used for Ca^2+^ imaging within 4 h after culture. Cells were incubated with Fura-2AM (10 μM, Abcam, UK) for 45 min. Cells were then washed 3 times and imaged in the loading buffer (containing 140 NaCl, 5 KCl, 2 CaCl_2_, 2 MgCl_2_, and 10 HEPES in mM, pH 7.4 with NaOH) as described 33. Ratiometric Ca^2+^ imaging was performed using a Nikon ECLIPSE Ti-S (Nikon, Japan) microscope equipped with a Polychrome V monochromator (Till Photonics, USA) and an Orca Flash 4.0 CCD camera (Hamamatsu, Japan). Images were captured and processed with MetaFluor software (Molecular Devices, USA). Ratiometric images were obtained with exposures of 0.5 ms at 340 nm and 0.3 ms at 380 nm excitation wavelengths, respectively. Representative Ca^2+^ imaging images were generated using ImageJ software (NIH, USA). A cell was considered responsive if the peak Ca^2+^ response is above 20% of the baseline according to our previous publication 34. WGA positively labeled (WGA^+^) DRG neurons were further selected for data analysis.

### Western blot

This method was used to examine TRPA1 protein expression changes in DRG after *Trpa1* gene knockdown. L3-L5 DRG samples were harvested 5 weeks after transfection. Samples were homogenized in RIPA buffer [50 mM Tris (pH7.4), 150 mM NaCl, 1% Triton X-100, 1% sodium deoxycholate, sodium orthovanadate, 0.1% SDS, EDTA, sodium fluoride, leupeptin, and 1 nM PMSF], then centrifuged at 12,000 rpm for 10 min at 4 °C and the supernatant was then collected. The protein concentration was determined using BCA method according to the kit's instruction (Thermo Fisher, USA) and 15 μg protein was loaded in each lane. Protein was loaded and separated by SDS-PAGE and electrophoretically transferred onto PVDF membranes. The membranes were blocked with 5% non-fat milk in TBST solution for 1 h at room temperature, and then the membranes were incubated with primary antibodies: anti-TPRA1 (1:500, #ACC-037, Alomone Labs, Israel) overnight at 4 °C. Subsequently, the immunoblots were incubated with the secondary antibody anti-rabbit IgG (1:2000, #7074, CST, USA) for 2 h at room temperature. β-actin (1:5000, #ab20272, Abcam, UK) was used as reference control. The gel images were captured by FluoChem R (Biotechne, USA). Quantitative analysis was performed with ImageJ (NIH, USA).

### Immunostaining

This method was used to identify the cellular distribution of IL-33-positive staining and quantify macrophages. The mice were deeply anesthetized with isoflurane, and were perfused through the ascending aorta with 0.9% saline followed by 4% fresh paraformaldehyde in 0.01 M PBS. After perfusion, the ipsilateral skin, L3-5 DRGs and spinal cord were removed and post-fixed in 4% paraformaldehyde for 4-6 h (4 °C) before transferring to 15% and 30% sucrose for dehydration. Samples embedded in a frozen microtome (Thermo NX50, Thermo Fisher, USA) were cut into frozen sections with the thickness of 12 μm for hindpaw skin, 8 µm for DRG and 20 μm for spinal cord. Then samples were mounted onto gelatin-coated glass slides for immunofluorescence. The tissues were first blocked with 5% donkey serum in TBST for 1 h at 37 °C, then incubated with the following primary antibodies at 4°C overnight: anti-IL33 (1:200, #AF3626, R&D Systems, USA), anti-keratin14 (1:100, #ab119695, Abcam, UK), anti-c-Fos (1:200, #2250, CST, USA), anti-Iba1 (1:500, #ab178846, Abcam, UK), anti-8-OHdG (1:800, #ab62623, Abcam, UK), anti-NeuN (1:300, #104224, Abcam, UK). After washing, the tissues were incubated with corresponding secondary antibodies (Cy3-, Cy5-, or FITC-conjugated) for 1 h at 37 °C after washing in the dark. Images were captured by Axio Imager M2 microscope (Zeiss, Germany).

### Flow cytometry

This method was used to quantify macrophage infiltrations in skin tissues. Ipsilateral hindpaw skin tissues were removed and transferred to digesting solution containing 1 mg/ml collagenase A and 2 mg/ml dispase in DMEM. Then the cells were incubated at 37°C with shaking for 1 h at 50 rpm. After removing the supernatant, the cells were washed twice with DMEM and PBS, and filtered through a 70 μm cell filter. 1×RBC lysis buffer (#C3702, Beyotime, China) was used to reduce RBC contamination. Surface expression of F4/80 and CD11b was analyzed by flow cytometry. The corresponding antibodies were added to 500 μl PBS containing 5% FBS, and incubate in the dark at 4°C for 1 h. The antibodies used were as follows: F4/80-PE (1:1000, #565410, BD, USA), CD11b-APC (1:1000, #562102, BD, USA). After staining, the cells were washed in PBS for 3 times. Flow cytometry events were acquired in a CytoFLEX S (Beckman Coulter, USA). Data were analyzed by CytExpert Software (Beckman Coulter, USA).

### qPCR

This method was used to examine gene expression changes. Total RNA from ipsilateral hind paw skin tissue was extracted by TRIzol reagent (Thermo Fisher, USA). 1,000 ng of total RNA was reversely transcribed with PrimeScript RT Reagent Kit (Takara Bio Inc., Japan) into cDNAs. qPCR was performed using TB Green Premix Ex Taq II (Takara Bio Inc, Japan) as the master kit with CFX96 Real-Time System (Bio-Rad Laboratories Inc., USA). β-actin gene (*Actb*) was used as an internal reference gene. Each reaction was performed in triplicates and normalized to* Actb* gene expression. The cycle threshold (CT) value of each well was deduced using CFX96 Real-Time System software and the average of the triplicates was calculated. The ^ΔΔ^CT method was utilized to determined relative quantification 35, 36. The detailed information regarding the sequences for the primer sequence (from 5ʹ to 3ʹ) was as follows: *Actb* forward: 5'-GTGCTATGTTGCTCTAGACTTCG-3', *Actb* reverse: 5'-ATGCCACAGGATTCCATACC-3'; *Il33* forward: 5'-CAGAAGACCAAAGAATTCTGCC-3', *Il33* reverse: 5'-CATGCTTGGTACCCGATTTTAG-3'; *St2* forward: 5'-TGACACCTTACAAAACCCGGA-3', *St2* reverse: 5'-AGGTCTCTCCCATAAATGCACA-3'.

### Raw264.7 cell culture and transwell migration assay

The transwell migration assay was used to determine chemotactic effect of IL-33/ST2 signaling on macrophage cell line. Raw264.7 murine macrophage cell line was purchased from Shanghai Academy of Life Sciences (Shanghai, China) and cultured in DMEM (Thermo Fisher, USA), supplemented with 10% FBS, 100 U/mL penicillin and 100 μg/mL streptomycin at 37 °C in a humidified atmosphere with 5% CO_2_. The chemotactic capability of IL-33 was determined using the Transwell plates (6.5 mm in diameter with 5 μm pore filters from Corning, USA). An amount of 5×10^4^ cells was suspended in 100 μl serum-free medium and were added to the upper well. Different doses of IL-33 or BSA containing medium was placed in the lower well of the Transwell plate. Following incubation for 6 h (37°C, 100% humidity, 5% CO_2_ in air), the migrated cells were fixed with 4% paraformaldehyde, stained with crystal violet (0.1%), and quantified in 5 randomly selected fields at a magnification of 200× for each test under Axio Observer. A1 microscope (Zeiss, Germany) using Zen software (Zeiss, Germany). Each experiment was performed in triplicate, and the data were averaged for statistical analysis.

### *In vitro* ROS assay

This method was used to evaluate ROS level in an *in vitro* setting. The detailed protocols of oxidative stress biomarkers examination were documented in our previous publication 37. Ipsilateral hindpaw skin tissues from each group were collected 1 or 3 d after model establishment. All samples were chopped and centrifuged and then the supernatant was collected for corresponding biochemical assays. The collected supernatants were then analyzed by means of commercially available kits for superoxide dismutase (SOD), reduced glutathione (GSH) from Nanjing Jiancheng Bioengineering Institute (China) and malondialdehyde (MDA) and hydrogen peroxide (H_2_O_2_) from Beyotime Biotechnology (China) according to the instructions 22. The absorbance was determined with SpectraMax M4 (Molecular Devices, USA) microplate reader at specified wavelength and the data was derived and analyzed using SoftMax Pro software (Molecular Devices, USA).

### *In vivo* ROS imaging

This method was used to monitor ROS changes in the incised skin tissue in an *in vivo* setting. 3 d after incision, the mice were injected (intravenously, i.v.) with chemiluminescence ROS probe L-012 (25 mg/kg, Tocris, USA), as described previously 24, 38. 5 min after the injection, mice were anesthetized with isoflurane, then placed on the stage of an IVIS Lumina LT *in vivo* imaging system (PerkinElmer, USA) and the fluorescence imaging was performed. The luminescence signal intensities were quantified with Living Image software (PerkinElmer, USA).

### Macrophage depletion

Clodronate-loaded liposome suspension (LIPOSOMA, the Netherlands, 5 mg/ml in 200 μl) was injected (i.p.) 1 d before and 4 d after the incision. Liposome was used as vehicle control. The efficiency of macrophage depletion was evaluated by immunostaining of the spleen and skin tissues with macrophage marker Iba-1.

### Macrophage adoptive transfer

This method was used to study the contribution of macrophages to the nocifensive behavior and oxidative stress in INC model mice. Thioglycollate-elicited macrophages (TPMs) were generated by injecting the mice (i.p.) with 1 ml 4 % Brewer's thioglycollate medium (Sigma, USA) for 3 consecutive days, adding up to 3 ml in total as previously reported 24. Mice were euthanized 24 h after the last injection. Peritoneal cavity cells were harvested by lavage, and cultured in DMEM plus 10% FBS, 100 U/mL penicillin and 100 μg/mL streptomycin. Then TPMs were incubated with Vybrant CM-Dil Cell-Labeling Solution (Thermo Fisher Scientific, USA) in the working solution for 5 min at 37°C, and then for an additional 15 min at 4°C. Cells were then washed 3 times with PBS and re-suspended as described before 39. The TPMs were injected (i.pl.) into hind paws of *St2*^-/-^ mice at a dose of 5×10^4^ cells per mouse 1 d after incision.

### AAV-PHP.S-mediated Trpa1 gene knockdown

This method was used to specifically knock down *Trpa1* gene expression in DRG neurons innervating the incised skin tissues. AAV-PHP.S-hSyn-shRNA (*Trpa1*)-EGFP, driven by the neuronal promoter hSyn, was used to specifically knockdown *Trpa1* gene in skin-innervating DRG neurons. The virus was constructed by BrainVTA (Wuhan, China). The virus was intraplantar (i.pl.) injected to the hind paw area that was going to be incised via a Hamilton micro syringe (10 μl volume, titer: 5.24×10^12^vg/ml) 5 weeks before incision. AAV-PHP.S-hSyn-scramble-EGFP was used as a negative control. 5 weeks later, the mice were subject to model establishment and behavioral assays.

### Wound healing assessment

For assessing wound closure following the plantar incision, each incision site was digitally photographed on 3, 4, and 7 days after the surgery. Changes in the wound areas were expressed as the width of the incised site as previously described 40.

### Raw data processing

The raw data were processed into respective presentation forms as in the figures of this study via GraphPad Prism 8 (GraphPad Software Inc., USA) and Adobe Illustrator CC 2017 (Adobe, USA).

### Statistical analysis

Statistical analysis was conducted using GraphPad Prism 8 (GraphPad Software Inc., USA). Data in bar graphs were expressed as mean ± SEM. Sample size was determined according to our recent studies that used similar outcome measures 24, 32, 41, 42. Student's *t*-test was used for comparisons between two groups. One-way or two-way ANOVA followed by Tukey's post hoc test was used for comparison among groups ≥ 3. ANOVA with repeated measures was taken if necessary. In cases when the data was non-normally distributed (tested by Kolmogorov-Smirnov test), a nonparametric test (e.g. Mann-Whitney test) was used for analysis. Comparison is considered significantly different if p < 0.05.

## Results

### IL-33 is produced from skin keratinocytes in a mouse model of INC pain

**Figure 1:**
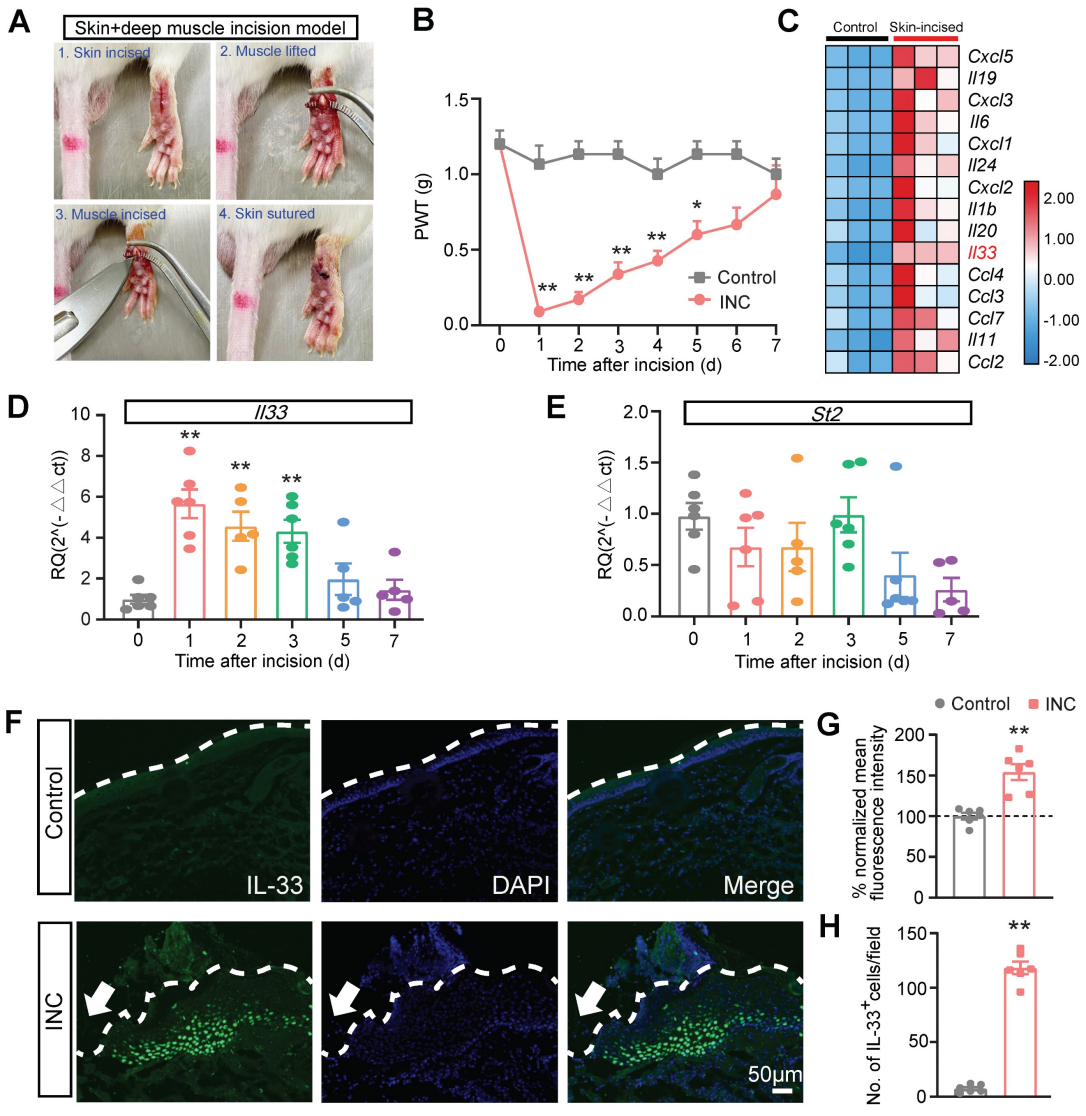
** IL-33 expression is upregulated in incised skin tissues of a mouse model of incisional pain.** (A) Representative pictures showing the procedures of establishing the mouse incisional (INC) pain model. (B) Paw withdraw threshold (PWT) determined by von Frey hair of control and INC pain group mice. n=6-7 mice/group. ^*^p<0.05 and ^**^p<0.01 vs. control group. (C) Heatmap showing the top 15 most highly up-regulated inflammatory cytokines or chemokines in the incised skin tissues of skin-incised group vs. control group. n=3 mice/group. (D&E) qPCR showing *Il33* and *St2* gene expression from day 0 to day 7 in INC pain model group. n=5-6 mice/group. ^**^p<0.01 vs. Day 0. (F) Representative immunofluorescence pictures showing IL-33 expression (in green) in skin tissues of control and INC pain model group mice. Purple color denotes DAPI staining. Dashed line indicates stratum corneum of epidermis. Scale bar indicates 50 μm. (G) Summary of normalized mean fluorescence intensity of IL-33 in control and INC group as in panel F. Control group value was taken as 100% and INC group was normalized. n=6 mice/group. (H) Summary of the No. of IL-33 positively stained (IL-33^+^) cells per observational field in control and INC group. n=6 mice/group. ^**^p<0.01 vs. control group. Two-way ANOVA followed with Tukey's post-hoc test was used for panel B. One-way ANOVA followed with Tukey's post-hoc test was used for panel D&E. Student's unpaired *t* test was used for panel G&H.

We established a mouse model of skin plus deep tissue incision-induced INC pain as previously described (Fig. 1A) 43. Compared with control group, INC pain group mice developed robust mechanical allodynia, manifested by the reduction of paw withdraw threshold (PWT), which persisted over 5 days and gradually returned back to normal after 7 days (Fig. 1B). In order to explore potential endogenous pain mediators in INC pain model mice, we downloaded a RNA-Sequencing dataset which examines gene expression changes in the skin of a wound healing mouse model 44. We examined the pro-inflammatory cytokine or chemokine genes involved in this model (Fig. 1C). We were especially interested in *Il33*, which has not been demonstrated in INC pain, but has been documented to contribute to some other pain conditions 18 (Fig. 1C). qPCR validated that *Il33* gene expression was indeed increased in skin of INC group mice on day 1 to 3 (Fig. 1D). In contrast, the expression of *St2*, the gene encoding specific receptor for IL-33, remained unchanged (Fig. 1E). We then performed immunostaining and found that the immunoreactivity of IL-33, as well as IL-33-positively stained (IL-33^+^) cells, were both significantly increased in skin tissues of INC pain group mice vs. control mice 1 day after incision (Fig. 1. F-H).

**Figure 2:**
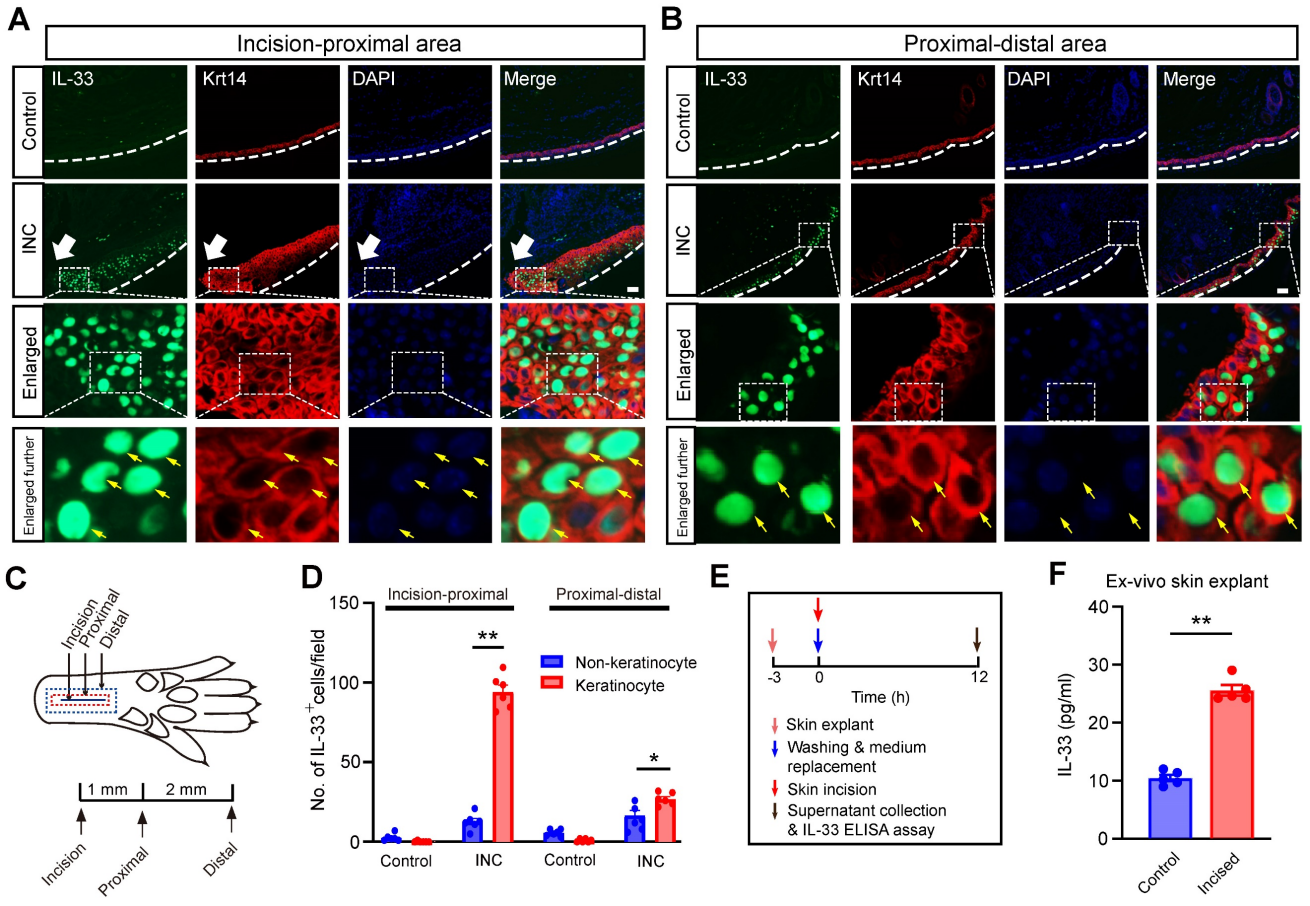
** IL-33 is produced from keratinocytes upon skin incision.** (A&B) Representative immunofluorescence pictures showing IL-33 (in green) co-expression with keratinocyte marker Krt14 (in red) in skin tissues from both control and INC pain model group mice. White arrow indicates the incision site. White line denotes stratum corneum of epidermis. The area covered by the white dashed box is further enlarged and shown in the lower panels as indicated. Yellow arrows indicate IL-33^+^ keratinocytes. Panel A was captured from the “proximal-incision area”, whereas panel B was from “distal-proximal area” as indicated in panel C. Scale bar: 50 μm. (C) Schematic picture showing the location of the observational field. Proximal is 1 mm apart from the incision, whereas distal is 3 mm apart from the incision, respectively. (D) Summary of IL-33^+^ staining in keratinocytes and non-keratinocytes in skin tissues of INC group mice. IL-33-positive cell was defined as having 2 characters simultaneously: 1. Being stained positive for IL-33; 2: Having a DAPI-labeled nucleus. The observation field is 650 μm in length and 520 μm in width. 6 sections pooled from 3 mice were included in each group. (E) Time points for *ex vivo* skin explant culture and IL-33 release assay. (F) IL-33 concentration in culture medium of control and incised group determined by ELISA. Medium was collected 12 h after incision was made in skin explant. 5 skin explants were included in each group and summarized. One-way ANOVA followed with Tukey's post-hoc test was used for panel D. Student's *t* test was used for panel F.

We then explored the cellular source for the increased IL-33 expression in skin from INC group mice. It is known that IL-33 can be produced from keratinocytes or macrophages upon skin injury or inflammation 21, 24. We first checked the skin isolated from incision to the proximal area, which includes the incised skin (Fig. 2A&C). Double immunofluorescence staining indicated that IL-33^+^ cells closely overlapped with cells co-stained with keratinocyte marker keratin 14, but not with macrophage marker Iba-1 (Fig. 2A & Supplementary Fig. 1). Skin samples isolated from proximal-distal area of the INC group mice showed similar co-staining pattern of IL-33 with keratinocytes (Fig. 2B). Statistics showed that IL-33^+^ cells predominantly co-stained with keratinocytes in the skin of INC group mice (Fig. 2D). Since IL-33 was predominantly produced by keratinocytes, we next wanted to know whether IL-33 could be released into extracellular space upon skin incision. We established an ex-vivo ear skin tissue culture system. We found IL-33 level was significantly increased in culture medium derived from ear skin explant receiving incisions vs. control group (Fig. 2E&F), suggesting an extracellular IL-33 release upon skin incision.

### IL-33/ST2 signaling contributes to mechanical hypersensitivity and gait impairments in INC pain model mice

**Figure 3:**
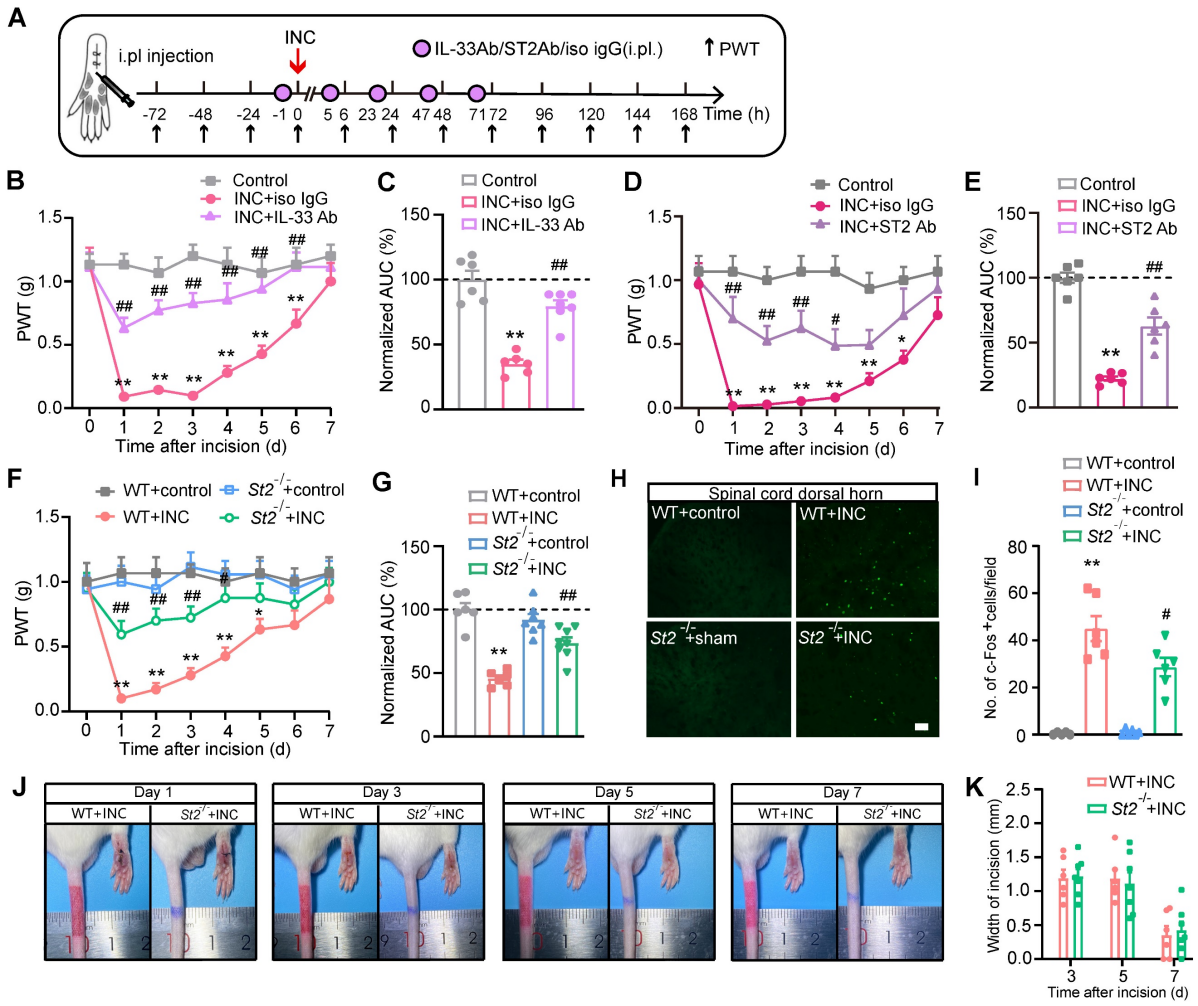
** IL-33/ST2 signaling mediates mechanical hypersensitivity of INC pain model mice.** (A) Experimental protocol denoting time points for the incision, treatment and behavioral observation. (B) Overlaid time courses showing PWT changes in control, INC+Iso IgG and INC+IL-33 neutralizing antibody (IL-33 Ab) groups. (C) Normalized area under the curve (AUC) analysis of each curve in panel B. (D) Overlaid time courses showing PWT changes in control, INC+Iso IgG and INC+ST2 neutralizing antibody (ST2 Ab) groups. (E) Normalized AUC analysis of each curve in panel D. (F) Overlaid time courses showing PWT changes in 4 groups of mice as indicated. (G) Normalized AUC analysis of each curve in panel F. (H) Representative pictures of c-Fos immunostaining in ipsilateral spinal cord dorsal horn of 4 groups of mice. Scale bar represents 100 μm. (I) Summarized data showing c-Fos^+^ cells per observational field of 4 groups. (J) Representative photos showing wound healing of WT+INC vs. St2^-/-^+INC group of mice on Day 1, 3, 5&7 after incision. (K) Summary of the width (in mm) of the incision on Day 3, 5&7 as in panel J. The suture was removed 1 day after the incision. ^**^p<0.01 vs. control group. ^#^p<0.05, ^##^p<0.01 vs. IL-33 Ab/ST2 Ab/WT+INC group as indicated. n=6 mice/group. Two-way ANOVA followed with Tukey's post-hoc test was used for panel B, D, F&K. One-way ANOVA followed with Tukey's post-hoc test was used for panel C, E, G&I.

We then examined whether IL-33 contributes to mechanical hypersensitivity of INC pain model mice. IL-33 neutralizing antibody or isotype control IgG was administered to mice 1 h before and 5, 23, 47 and 71 h after INC pain model establishment (Fig. 3A). Neutralizing IL-33 significantly ameliorated mechanical allodynia of INC pain model mice (Fig. 3B). Area under the curve (AUC) indicated reduced mechanical allodynia in INC+IL-33 Ab group vs. INC+Iso IgG group (Fig. 3C). Furthermore, injecting excessive recombinant mouse IL-33 (rmIL-33) to the incised skin worsened mechanical allodynia of INC model mice vs. vehicle injection until Day7 (Supplementary Fig. 2A-C).

Next, we explored whether ST2 was involved in mechanical allodynia of INC pain model mice using ST2 neutralizing antibody. ST2 neutralizing antibody was administered at the same time points as IL-33 neutralizing antibody (Fig. 3A). Similarly, neutralization of ST2 significantly ameliorated mechanical allodynia of INC pain model mice (Fig. 3D&E). We further studied the involvement of ST2 by means of *St2* gene deficient (*St2*^ -/-^) mice. *St2*^ -/-^ mice showed similar paw withdraw thresholds with wild type (WT) controls under normal condition (Fig. 3F). However, after incision, *St2*^ -/-^mice (*St2*^-/-^+INC) showed significantly improved mechanical allodynia compared with WT mice (*St2*^-/-^+INC vs. WT+INC) (Fig. 3F&G). Additionally, *St2*^ -/-^mice did not exhibit any deficit in locomotor activity compared with WT control (Supplementary Fig. 3A&B). The c-Fos expression in spinal cord dorsal horn (SCDH) serves as an reliable neuronal marker for pain signals 45. We found that c-Fos expression was significantly increased in WT+INC group compared with WT+control group on Day 3 after incision. In contrast, c-Fos expression in SCDH was reduced in* St2*^-/-^+INC group (Fig. 3H&I). Moreover, *St2*^-/-^ mice did not exhibit any deficit in wound healing capability compared with WT mice after skin incision (Fig. 3J&K).

**Figure 4:**
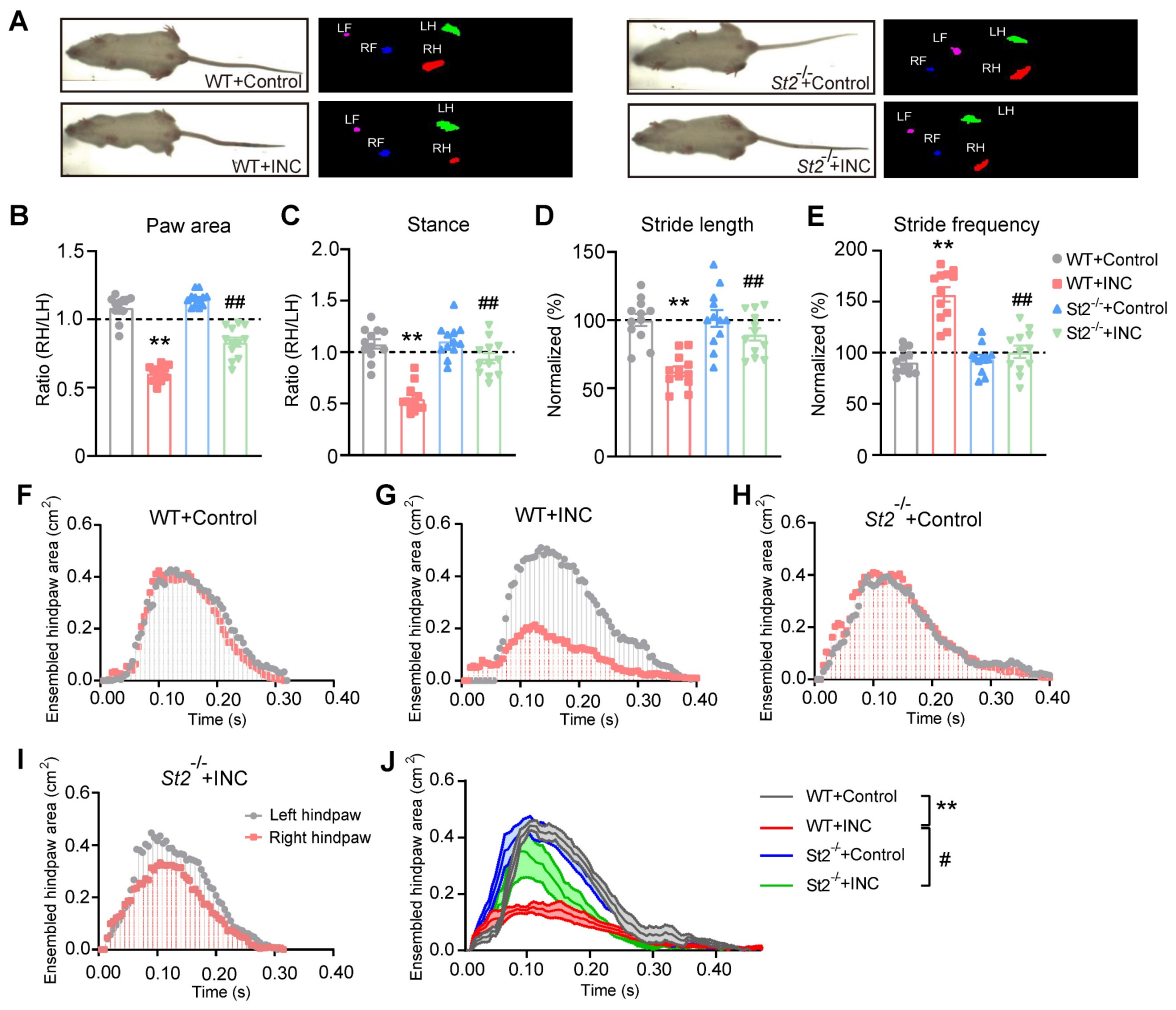
***St2*^-/-^ mice showed improvement in gait impairments caused by skin and deep tissue incision.** (A) Representative pictures showing 4 groups of mice monitored and analyzed by the gait analyzing system. Left pictures: instant recordings of mice. Right pictures: corresponding paw area analysis. Green color denotes left hind paw (LH). Red color denotes right hind paw (RH). (B-E) Summary of paw area ratio (RH/ LH), stance ratio (RH/LH), stride length and frequency of 4 groups of mice 1 day after incision/sham operation. n=11-12 mice/group. (F-I) Representative traces showing dynamic change of ensemble hind paw area of the right vs. left hind paw from 4 groups of mice 1 day after incision. (J) Summary of the overlaid dynamic changes in ensemble hind paw area (right) obtained from all 4 groups as in panel F-I. n=11-12 mice/group. ^**^p<0.01 vs. WT+Control group. ^##^p<0.01, ^#^p<0.05 vs. WT+INC group. One-way ANOVA followed with Tukey's post-hoc test was used for panels B-E&J.

INC pain can become more severe while moving, which limits the patients' movements, daily activities and can even produce impacts on their abilities to participate in postsurgical rehabilitation 46-48. We then examined whether movement can produce pain-related gait impairment in INC pain model mice. Gait analysis indicated that INC pain model mice showed significant gait impairments when walking on a treadmill compared with control mice in terms of paw area, stance, stride length and frequency (Fig. 4A-E) 1 day after incision. In contrast, *St2*^-/-^ mice showed remarkably improved gait parameters compared with WT mice when incision was made (Fig. 4A-E). A more detailed spatiotemporal characterization of footprints further uncovered that INC pain model mice (WT+INC) developed abnormal dynamic changes in ensemble area of the right (ipsilateral) hind paw vs. WT+Control group mice, whereas *St2*^-/-^+INC group mice exhibited significantly improved ensemble area vs. WT+INC group mice (Fig. 4F-J). These results indicate that IL-33/ST2 signaling contributes to mechanical hypersensitivity and gait impairments of INC pain model mice.

### IL-33/ST2 signaling is involved in oxidative stress modulation and ROS production in skin tissues of INC pain model mice

**Figure 5:**
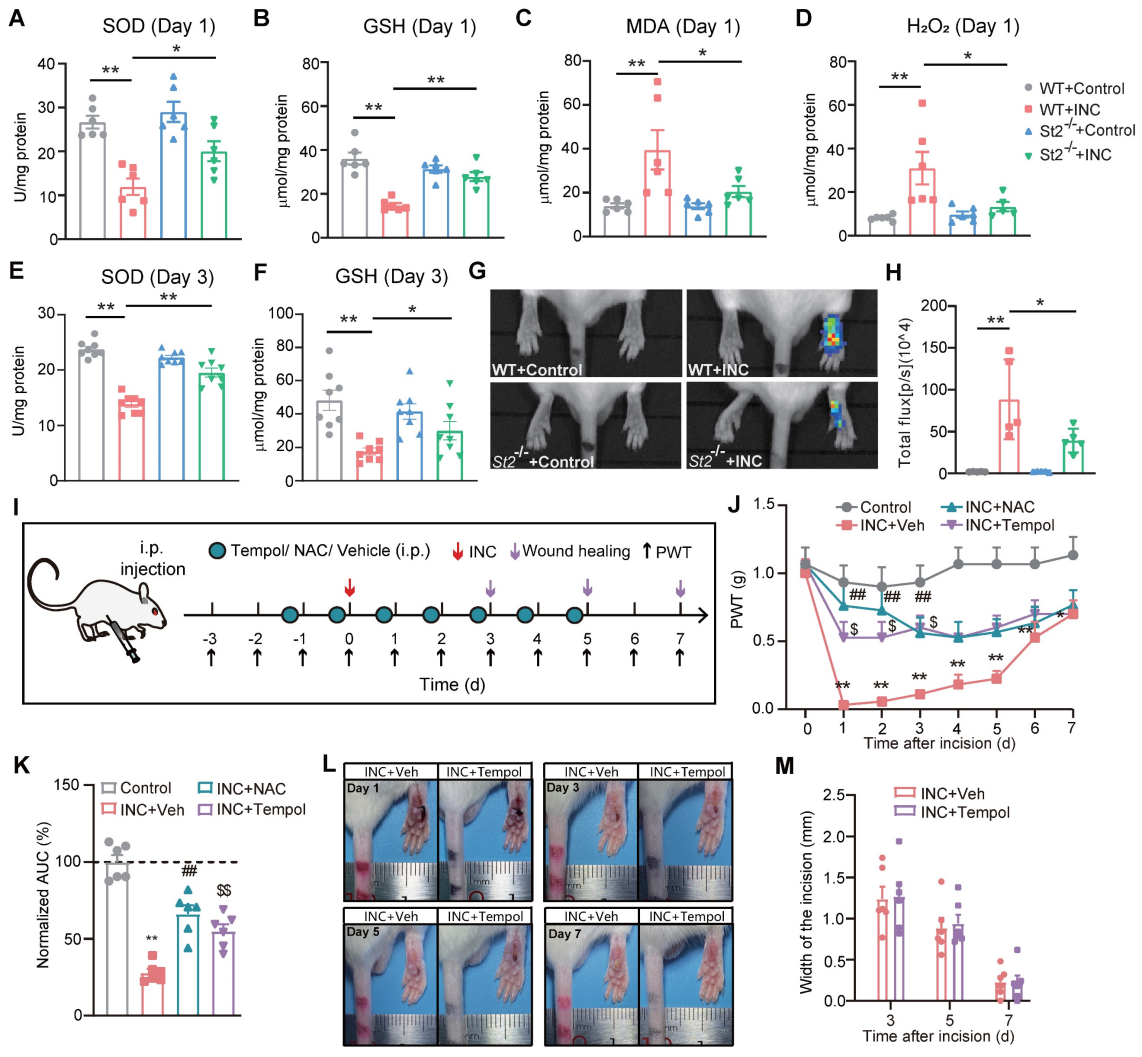
** ST2 is involved in oxidative stress modulation and promotes ROS production in skin tissues of INC pain model mice that contributes to mechanical allodynia.** (A-D) Summary of SOD activity, GSH-Px, MDA and H_2_O_2_ levels in WT and ST2^-/-^ mice 1 day after incision/sham operation. (E&F) Summary of SOD activities and GSH-Px level in WT and ST2^-/-^ mice 3 days after incision/sham operation. (G) *In vivo* imaging monitoring ROS level in the incised hind paw of 4 groups of mice 3 days after incision/sham operation. (H) Summary of the total L-012 chemiluminescent flux in the incised hind paw as in panel G. ^**^p<0.01, ^*^p<0.05. (I) Protocol showing time points for drug application, model establishment, wound healing assessment and behavioral assay. (J) Overlaid time courses showing PWT changes in Control, INC+Veh, INC+NAC and INC+Tempol groups of mice. (K) Summary of normalized AUC analysis as in panel J. (L) Representative photos showing the wound healing of INC model mice receiving vehicle or Tempol treatment on Day 1, 3, 5 and 7 after incision. ^##^p<0.01, ^$^p<0.05, ^$$^p<0.01 vs. INC+Veh group. (M) Summary of the width of the incision of two groups of mice as in panel L. n=5-6 mice/group. Two-way ANOVA with Tukey's post-hoc test was used for panel J&M. One-way ANOVA followed with Tukey's post-hoc test was used for others.

We next studied the mechanism underlying how IL-33/ST2 contributes to mechanical hypersensitivity in INC pain model mice. It is known that endogenous ROS products resulted from oxidative stress play a critical role in mediating pain 31, 49, 50. ROS products have been reported to be up-regulated in skin and muscle tissues of INC pain model animals 51. Our recent work indicates that IL-33/ST2 signaling contributes to ROS overproduction in the inflamed joints of a mouse gout arthritis model 24. These findings inspired us to wonder whether IL-33/ST2 might regulate oxidative stress status and ROS production in INC pain model mice. Biochemistry analysis revealed that the activities of SOD and GSH-Px, two major antioxidant enzymes involved in oxidative stress, were both reduced in skin tissues of WT+INC pain model mice vs. WT+Control group 1 day after incision, whereas *St2*^-/-^+INC group significantly reversed the downregulated activities of SOD and GSH-Px vs. WT+INC group (Fig. 5A&B). The level of malondialdehyde (MDA), a lipid peroxidation product, and the level of H_2_O_2_, an endogenous ROS product, were both significantly increased in the skin of INC model mice 1 day after incision, whereas *St2*^-/-^+INC group significantly reversed the upregulated levels of MDA and H_2_O_2_ vs. WT+INC group (Fig. 5C&D). Moreover, the activities of SOD and GSH-Px remained downregulated 3 days after incision in WT+INC group, which was significantly reversed in *St2*^-/-^+INC group (Fig. 5E&F).

We proceeded to monitor endogenous ROS production in the incised area via a noninvasive *in vivo* imaging approach with chemiluminescent probe L-012 3 days after incision. WT+INC group showed significantly upregulated chemiluminescent signal in the incised area vs. Control group, reflecting a rise in ROS production *in vivo* (Fig. 5G&H). However, the chemiluminescent signal was much abrogated in *St2*^-/-^+INC group mice (Fig. 5G&H). These results demonstrate that oxidative stress occurs in skin tissues of INC pain model mice and IL-33/ST2 contributes to oxidative stress modulation and ROS production.

We then investigated whether the upregulated oxidative stress may be involved in mechanical hypersensitivity of INC model mice. To this end, we used two well-known ROS scavengers, Tempol and NAC, to decrease ROS overproduction. NAC is a precursor of intracellular cysteine and of reduced glutathione (GSH) and serves as an inhibitor of ROS production. Tempol is a SOD mimetic and free radical scavenger. We successfully used these two reagents to reduce ROS overproduction in the inflamed joint tissues in our previous study 37. Therefore, we continued to use these two reagents in our following study to reduce ROS overproduction in the incised skin tissues. Tempol (200 mg/kg) or NAC (200 mg/kg) was administered (i.p.) to mice every day after incision as indicated in Fig. 5I. Tempol or NAC treatment could each significantly ameliorate mechanical allodynia of INC pain model mice compared with vehicle treated group (INC+Veh) (Fig. 5J&K). We further evaluated the potential effects of Tempol or NAC treatment on skin wound healing of INC pain model mice. Results indicated that Tempol or NAC treatment had no significant impact on wound healing of INC pain model mice (Fig. 5L&M). These data demonstrate that ROS contribute to mechanical hypersensitivity of INC model mice.

Oxidative stress can activate a variety of transcription factors, resulting in the upregulation of inflammatory mediators 52. The gene expressions of certain pro-inflammatory cytokines, e.g. *Il1b*, *Il6*, *Cxcl1* and *Ccl2* were significantly increased in the incised skin tissues of INC pain model mice, but were significantly reduced in *St2*^-/-^ mice (Supplementary Fig. 4A). However, *St2* was not involved in anti-inflammatory gene expression modulation (Supplementary Fig. 4B).

### IL-33/ST2 promotes macrophage infiltration in incised skin to generate ROS that contribute to mechanical hypersensitivity of INC model mice

**Figure 6:**
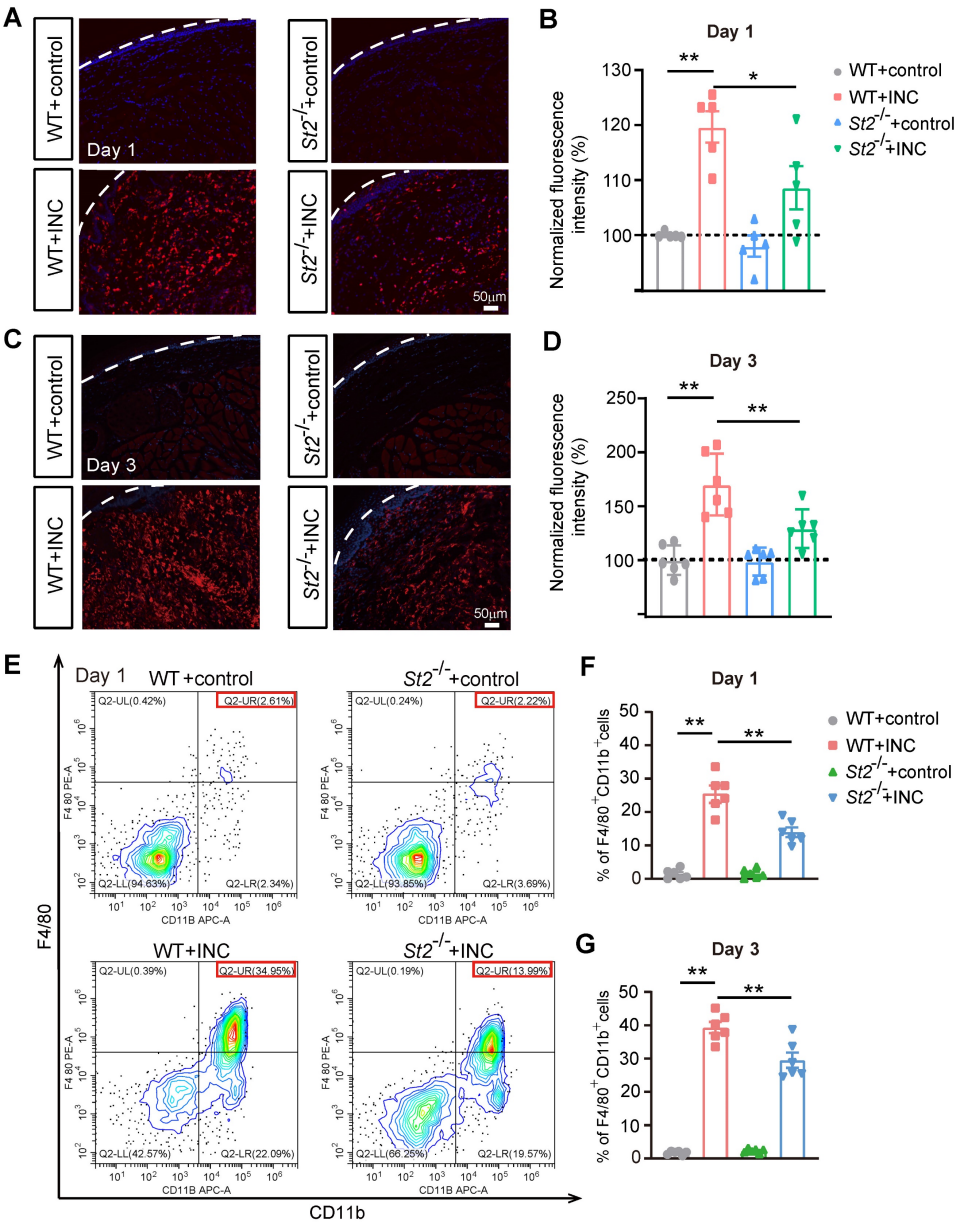
***St2*^-/-^ mice showed reduced macrophage infiltration in the incised skin tissues of INC pain model mice.** (A) Representative photos showing Iba-1 immunostaining (in red) in skin tissues of 4 groups of mice one day after incision/sham operation. Purple: DAPI staining. Dashed line indicates stratum corneum of epidermis. (B) Summary of normalized Iba-1 fluorescence intensity as in panel A. (C) Representative photos showing Iba-1 immunostaining in skin tissues of 4 groups of mice 3 days after incision/sham operation. (D) Summary of normalized Iba-1 fluorescence intensity as in panel C. Scale bar=50 μm. (E) Representative flow cytometry analysis of macrophages expressing F4/80 and CD11b in cell suspensions isolated from skin tissues of 4 groups of mice 1 day after incision/sham operation. F4/80 and CD11b double positive cell population was judged as macrophages. A total number of 10,000 cells were collected and analyzed in each assay. The red box indicates the % of F4/80^+^&CD11b^+^ cells among total cells in the panel. (F&G) Summary of % of F4/80^+^&CD11b^+^ cells 1 (F) or 3 days (G) in skin tissues of 4 groups after incision or sham operation as in panel E. n=5-6 mice/group. ^**^p<0.01, ^*^p<0.05. One-way ANOVA followed with Tukey's post-hoc test was used.

We next explored the mechanism underlying how IL-33/ST2 may promote oxidative stress and ROS production in INC model mice. Macrophage is a prominent inflammatory cell that infiltrates in wounds 53. Infiltrated macrophages generate ROS via oxidative burst to eliminate invasive microorganisms 54. Immunostaining revealed that Iba-1 positively stained macrophages were significantly increased in the incised skin on Day 1 and Day 3 of INC pain model mice (WT+INC) compared with WT+Control group (Fig. 6A-D). In contrast, *St2*^-/-^+INC group showed significantly reduced Iba-1 staining intensity vs. WT+INC group (Fig. 6A-D). Furthermore, quantification using flow cytometry showed that the % of F4/80^+^/CD11b^+^ macrophages was significantly increased in skin samples from INC pain model group on Day 1 and Day 3, whereas *St2*^-/-^+INC group showed significantly reduced % of F4/80^+^/CD11b^+^ macrophages vs. WT+INC group (Fig. 6E-G).

To further confirm that IL-33 can indeed promote macrophage infiltration via its specific receptor ST2, we tested macrophage chemoattraction towards conditioned medium containing rmIL-33 or vehicle (BSA) via transwell migration assay (Supplementary Fig. 5A). Conditioned medium containing rmIL-33 could dose dependently induce chemotactic effect on Raw 264.7 cells (Supplementary Fig. 5B&C). The chemotactic effect of rmIL-33 was near completely abolished by co-applying ST2 neutralizing antibody in the medium (Supplementary Fig. 5D-F). Interestingly, incubation with LPS in conditioned medium further enhanced rmIL-33-induced chemotactic effect on Raw 264.7 cells (Supplementary Fig. 5G-I).

**Figure 7:**
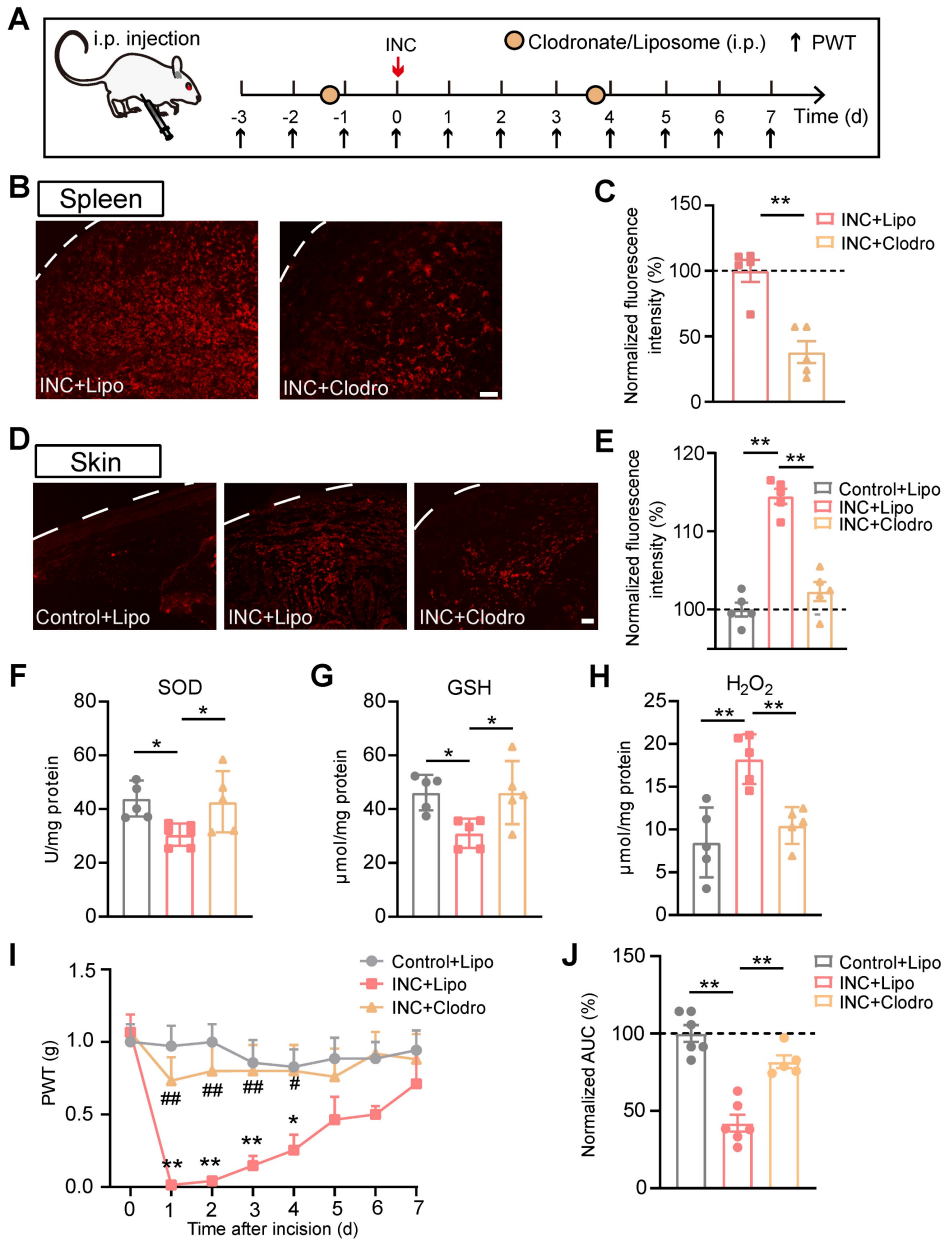
** Infiltrated macrophages in incised skin tissues contribute to ROS production and mechanical hypersensitivity of INC pain model mice.** (A) Experimental protocol showing time points for Clodronate/Liposome (i.p.) application, model establishment and behavioral assays. (B) Representative pictures showing Iba-1 immunostaining in spleen of INC model mice treated with liposome (Lipo) or clodronate (Clodro). Scale bar=100 μm. (C) Summary of normalized Iba-1fluorescence intensity (%) in INC+Lipo and INC+Clodro groups. (D) Representative pictures showing Iba-1 immunostaining in skin of control group mice receiving liposome and INC group mice receiving liposome/clodronate treatment. Scale bar=100 μm. (E) Summary of normalized Iba-1fluorescence intensity (%) in Control+Lipo, INC+Lipo and INC+Clodro groups as in panel D. (F-H) Summary of SOD activity, GSH-Px and H_2_O_2_ levels in skin tissues from 3 groups as indicated. (I) Overlaid time course showing PWT changes in 3 groups of mice. ^**^p<0.01, ^*^p<0.05 vs. Control+Lipo group. ^##^p<0.01, ^#^p<0.05 vs. INC+Lipo group. (J) Normalized AUC analysis (%) of curves as shown in panel I. n=5-6 mice/group. ^**^p<0.01, ^*^p<0.05. Student's unpaired *t* test for panel C. One-way ANOVA followed with Tukey's post-hoc test for panels E, F, G, H&J. Two-way ANOVA followed with Tukey's post-hoc test for panel I.

These above results indicate that IL-33/ST2 signaling mediates macrophage infiltration and ROS generation in skin tissues of INC pain model mice. However, it remains unknown whether macrophages may serve as a potential cellular source for ROS generation in model mice. To address this issue, we depleted macrophages *in vivo* with clodronate-containing liposomes (Fig. 7A). Systematic administration of clodronate (Clodro, 1 mg/100 μl, i.p.) successfully depleted macrophages as shown by the significantly reduced Iba-1staining in spleen of INC pain model mice compared with liposome (Lipo)-treated group (Fig. 7B&C). More importantly, macrophage infiltration in incised skin was significantly reduced by clodronate administration as well (Fig. 7D&E). These results demonstrate successful depletion of macrophages via clodronate in INC pain model mice. Clodronate-mediated macrophage depletion significantly reversed the oxidative stress imbalance by replenishing SOD and GSH-Px activities and reduced H_2_O_2_ overproduction in skin tissues of INC pain model mice (Fig. 7F-H). Additionally, macrophage depletion attenuated mechanical allodynia of INC pain model mice (Fig. 7I&J).

**Figure 8:**
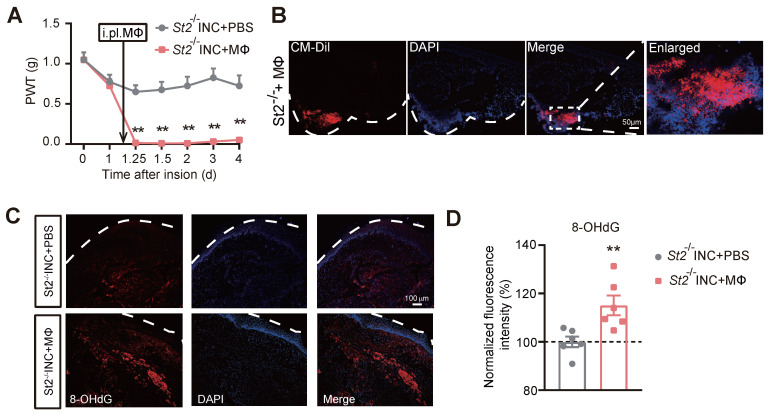
** The transfer of excessive wild type macrophages reproduces persistent mechanical hypersensitivity in *St2*^-/-^ mice underwent incision.** (A) Overlaid time courses showing PWT changes of *St2*^-/-^ mice underwent incision (*St2*^-/-^ INC) with wild type macrophages (MΦ) or PBS being injected into the incised skin tissue. Black arrow indicates the time point of injection. (B) Immunostaining showing the continued presence of injected macrophages (labeled with CM-DiI in red color) in the incised skin tissue 4 days after transfer. White dashed line indicates stratum corneum of epidermis. White dashed area is enlarged and shown on the right panel. Scale bar indicates 50 μm. (C) Representative photos showing the immunostaining of 8-OHdG, a marker for oxidative stress-induced cellular damage, in *St2*^-/-^ mice with incision that were injected with macrophages or PBS. (D) Summary of the normalized 8-OHdG fluorescence intensity (%) as in panel C. n=6 mice/group. Two-way ANOVA followed with Tukey's post-hoc test for panel A. Student's unpaired *t* test for panel D.^ **^p<0.01 vs. *St2*^-/-^ INC+PBS group.

To confirm whether macrophages are sufficient enough to produce pain in INC pain model mice, we carried out adoptive transfer experiment of injecting excessive WT macrophages into *St2*^-/-^ mice 1 day after skin incision (Fig. 8A). The deficiency in mechanical hypersensitivity of *St2*^-/-^ mice after incision was rescued by transplantation of WT macrophages (Fig. 8A). As expected, the transplanted macrophages, which were pre-labelled by CM-DiI, still remained in the injected hind paw skin tissues of *St2*^-/-^ mice 4 days after the transfer (Fig. 8B). Strikingly, transfer of excessive macrophage resulted in a significant increase of oxidative stress-induced DNA damage in skin tissues monitored with marker 8-hydroxy-2'-deoxyguanosine (8-OHdG) (Fig. 8C&D). Collectively, these results demonstrate that IL-33/ST2 signaling promotes macrophage infiltration to generate ROS that contribute to mechanical hypersensitivity of INC model mice.

### TRPA1 channel is functionally up-regulated in sensory neurons innervating incisional skin tissues and contributes to incision-induced mechanical hypersensitivity

**Figure 9:**
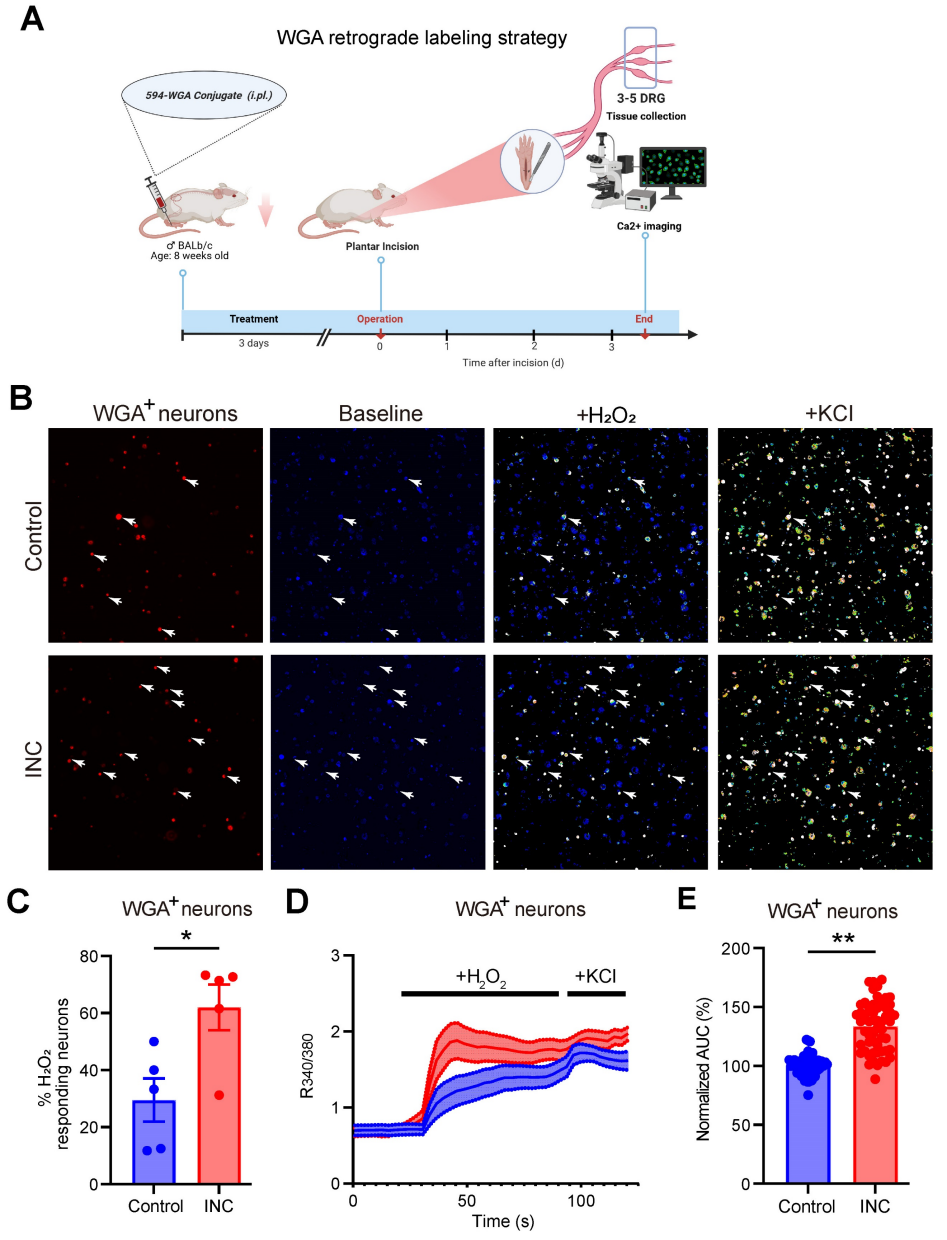
** TRPA1 channel function is increased in DRG neurons innervating the incised tissues of INC pain model mice.** (A) Cartoon showing the design and workflow of Ca^2+^ imaging experiments. The retrograde labeling dye WGA was used to label incisional site-innervating sensory neurons. The schematic picture was created with Biorender. (B) Representative pictures showing the cellular Ca2+ responses under control and in response to H_2_O_2_ (500 μM) and KCl (40 mM) challenges in DRG neurons isolated from control and INC pain model group mice. WGA-labeled (WGA^+^) neurons were indicated by red color and shown on the left panel. The white arrows indicate WGA^+^ neurons that responded to both H_2_O_2_ and KCl. (C) Summary of percentage of WGA^+^ DRG neurons that respond to H_2_O_2_ from control or INC pain model group mice. n = 5 tests/group. (D) Averaged Ca^2+^ responses of WGA^+^ DRG neurons elicited by H_2_O_2_ and KCl in control and INC pain model group. n > 20 cells/group. (E) Summary of normalized AUC of Ca^2+^ transients in response to H_2_O_2_ of INC and control group DRG neurons. n=55 cells/group, which was derived from 3-4 mice. Student's unpaired *t* test for panels C&E.

It is known that ROS products, e.g. H_2_O_2_, can act upon nociceptive TRPA1 channel in sensory neurons to elicit pain 55-57. TRPA1 channel function in sensory neurons can be up-regulated under certain pain conditions or by inflammatory signals, thus resulting in peripheral sensitization 24, 58-60. We aimed to explore whether TRPA1 channel activity could be functionally up-regulated in sensory neurons of INC pain model mice. The retrograde tracer WGA was injected into the incisional site 3 days before incision to specifically label DRG neurons innervating the incisional site (Fig. 9A). 3 days after incision, ipsilateral L3-L5 DRG were collected from control and INC pain model mice and subject to Ca^2+^ imaging. TRPA1 was stimulated by applying the endogenous TRPA1 channel agonist H_2_O_2_ (500 μM). WGA positively labelled (WGA^+^) DRG neurons were picked for further analysis. We found that the % of WGA^+^ DRG neurons responding to H_2_O_2_ challenge was significantly higher in INC group vs. control group (Fig.9B&C). Additionally, H_2_O_2_ elicited stronger Ca^2+^ responses in WGA^+^ DRG neurons from INC group than from control group (Fig. 9D&E). These results suggest that TRPA1 channel activity is functionally up-regulated in DRG neurons innervating incisional site.

**Figure 10:**
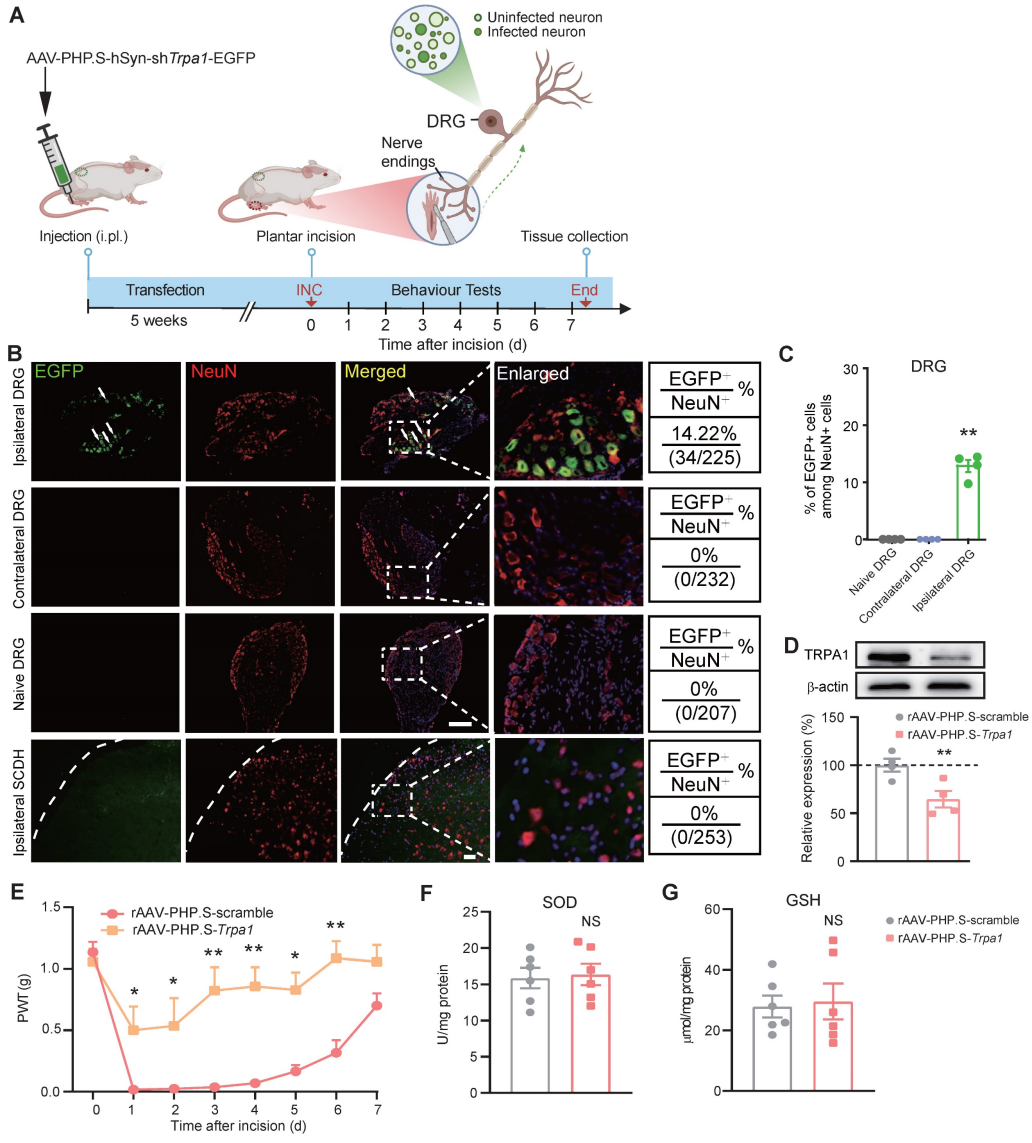
** TRPA1 channel innervating the incised tissues mediates mechanical allodynia of INC pain model mice.** (A) Cartoon showing the design of AAV-PHP.S that contains shRNA against *Trpa1* gene and is driven by neuron specific promoter hSyn. AAV-PHP.S.-hSyn-sh*Trpa1*-EGFP or scramble control virus was injected (i.pl.) into hind paw to transfect incisional area-innervating DRG neurons via its retrograde transportation property. The schematic picture was created with Biorender. (B) EGFP fluorescence of ipsilateral/contralateral L3-L5 DRG and ipsilateral spinal cord dorsal horn (SCDH). Scale bar indicates 100 μm. (C) Summary of EGFP^+^ cells among NeuN^+^ cells in ipsilateral DRG. NeuN: a neuron specific marker labeling DRG neurons. ^**^p<0.01 vs. Naïve DRG or contralateral DRG group. (D) Western blot showing TRPA1 protein expression in ipsilateral L3-L5 DRG of mice treated with AAV-PHP.S-scramble or AAV-PHP.S-sh*Trpa1*. n=4 mice/group. (E) Comparison of PWT of AAV-PHP.S-scramble- or AAV-PHP.S-sh*Trpa1*-treated group of mice upon incision. n=6-7 mice/group. ^**^p<0.01, ^*^p<0.05 vs. AAV-PHP.S-scramble-treated group. (F&G) SOD and GSH level in the incised tissues of AAV-PHP.S-scramble- or AAV-PHP.S-sh*Trpa1*-treated mice. NS: no significance vs. AAV-PHP.S-scramble group. One-way ANOVA followed with Tukey's post-hoc test for panel C. Student's unpaired *t* test for panel D, F&G. Two-way ANOVA followed with Tukey's post-hoc test for panel E.

We next examined whether targeted knockdown of *Trpa1* gene in DRG neurons innervating the incisional site would ameliorate INC-induced pain. To this end, we utilized AAV-PHP.S, which is designed as a capsid subtype specific for peripheral neuron transfection and possesses retrograde transfection property 61, 62 (Fig. 10A). AAV-PHP.S-hSyn-sh*Trpa1*-EGFP or AAV-PHP.S-hSyn-Scramble-EGFP was injected (i.pl.) into the incisional site 5 weeks before incision. The specificity of AAV-PHP.S-mediated transfection was verified by EGFP expression in ipsilateral L3-L5 DRG neurons but not in contralateral DRG neurons or spinal cord (Fig. 10B&C). The effectiveness of AAV-PHP.S-mediated gene knockdown was further verified by a significant reduction in TRPA1 protein expression in ipsilateral DRG by AAV-PHP.S-*Trpa1* compared with scramble-treated group (Fig. 10D). Specific *Trpa1* gene knockdown in DRG neurons innervating the incisional site did not affect basal mechanical pain threshold, but significantly alleviated mechanical allodynia of INC pain model mice after incision was made (Fig. 10E). Moreover, *Trpa1* gene knockdown in DRG neurons innervating the incisional site did not affect oxidative stress status as well as *Il33*/*ST2* gene expression in incised tissues (Fig. 10F&G, Supplementary Fig. 6A&B). These results indicate that ROS activates TRPA1 expressed in nociceptive sensory neurons innervating the incisional site to produce incisional pain.

## Discussion

IL-33 belongs to IL-1 family and is involved in both innate and adaptive immunity 16. At resting state, it is constitutively expressed in nuclei of a number of types of cells, particularly in those cells for maintaining barriers 63. When inflammation, injury or infection occurs, IL-33 functions as an alarmin to immune system and its expression is up-regulated and promptly released to the outside of the cell 64. In addition to its role as an alarmin, growing evidence indicates a critical role of IL-33 in many pain conditions 18, 19. Here we found that IL-33 expression to be significantly upregulated in the incisional skin tissues of INC pain model mice. Keratinocytes are known as a cellular source of IL-33 production in skin upon inflammation or injury insult 21, 22, 65. Our immunostaining results further indicated that IL-33 was predominantly expressed in the epidermis and exclusively expressed in skin keratinocytes. We examined the incised skin tissues collected from different areas around the incision. Results showed that IL-33 was highly expressed in both tissues adjacent to the incision and tissues surrounding the incision, indicating IL-33 expression was stimulated in a wide range of area surrounding the incision. It is known that IL-33 can be released from the nuclei of producing cells to extracellular space to serve as an alarmin and exerts effects on inflammatory cells after tissue injury or cellular damage 66. We found that tissue incision could promote IL-33 release into culture medium in mouse skin tissue explant culture, an indication of IL-33 extracellular release upon incision. Based upon these findings, we postulate that the released IL-33 from keratinocytes upon skin incision serves as an extracellular signal to communicate with other immune cells via receptor ST2, thus initiating inflammation and pain after incision.

Our immunostaining indicated that Iba-1^+^ macrophages were significantly increased in incisional skin of model mice, and reduced in St2^-/-^ mice. Similar results were also obtained by flow cytometry, which showed that F4/80^+^/CD11b^+^ macrophages were increased in skin of INC pain model mice, and decreased in St2^-/-^ mice. These results in all indicate that IL-33/ST2 signaling may regulate macrophage recruitment in incisional skin of model mice. ST2 receptor has been reported to be expressed in macrophages 67. Moreover, IL-33 can promote macrophage infiltration in tumors via ST2-dependent mechanism 68, 69. To further show that IL-33 can directly induce macrophage infiltration, we tested the chemotactic effect of IL-33 on RAW264.7 cells via transwell migration assay. The result showed that direct application of IL-33 can produce dose-dependent chemotactic effect on Raw264.7 cells via ST2-dependent mechanism, a result consistent with a previous study 69. Based upon this observation, we further demonstrated that IL-33 exerted significantly enhanced chemotactic effect upon RAW264.7 cells incubated with LPS. Studies have demonstrated that LPS is present in patients' blood samples after surgery, although its level is variable among patients 70-72. The presence of LPS after surgical procedure may likely enhance ST2 expression in macrophages, resulting in enhanced macrophage recruitment by IL-33 in the incisional area. This highlights the potential translational significance of our finding.

Macrophages oftentimes are among the first type of immune cells encountering the invading pathogens 73. Macrophages are known to release ROS via oxidative burst to exert antimicrobial effect as well as redox-regulation of immune signaling and initiation of inflammasome activation 54, 73. Here, we observed that ROS were significantly elevated in the incisional skin of INC pain model mice. By means of *in vivo* ROS imaging, we were able to observe ROS were upregulated in incisional skin of live animals, a result consistent with a previous study using a rat INC pain model 51. To further learn the cellular source of ROS, we depleted macrophages by systematic injection of a macrophage toxin clodronate. Depleting macrophages remarkably attenuated the up-regulated ROS levels in incisional skin of model mice. But it should be noted that neutrophils are also immune cells infiltrated in the incisional skin 10. Infiltrated neutrophils also produce ROS to contribute to the killing of microorganisms and regulation of inflammatory response 24. Thus, it remains likely that neutrophils may also contribute to ROS generation in the incisional skin of model mice. Besides, activated macrophages can release neutrophil chemoattractant, including CXCL1/CXCL2, to attract neutrophils 74. Thus, the depletion of macrophages by clodronate may resulted in less neutrophil infiltration and thus less ROS generation. Therefore, the exact cellular source of ROS in incisional skin still remains ambiguous at this moment, which needs further investigation. But our results clearly demonstrate that infiltrated macrophages constitute an important mechanism for ROS generation, either directly or indirectly, in incisional skin of model mice.

To further explore the potential molecular targets of ROS on nociceptive neurons, we focused on nociceptive TRPA1 channel. It is well established that TRPA1 channel serves as an oxidative stress sensor that can be activated by ROS or lipid peroxidation products to initiate pain 31, 55. Previous studies suggest that pharmacological blocking TRPA1 relieved thermal and mechanical pain sensitivity as well as pain-related guarding behavior in rat INC pain models 51, 75, 76. However, another study demonstrates that TRPA1 was not involved in nocifensive behavior of a mouse model of INC pain using *Trpa1* global knockout mice 25. It is well known that global gene knockout animals may display genetic compensation that could mask phenotypic differences 77. We thus utilized serotype AAV-PHP.S-mediated gene knockdown strategy, which exhibited specificity for peripheral neuron and retrograde transportation property 61, 78, to knockdown *Trpa1* expression specifically in DRG neurons innervating the incisional skin tissue. Our results showed that specific *Trpa1* knockdown in incisional skin-innervating DRG neurons effectively alleviated mechanical allodynia of INC pain model mice. Furthermore, with the aid of the retrograde labeling technique, we were able to find that the endogenous ROS product H_2_O_2_ can produce stronger TRPA1 channel activation in DRG neurons innervating the incisional area. We noticed that a previous study reported TRPA1 was not functionally up-regulated in retrograde labeled DRG neurons in skin-only incisional model mice 25. However, it should be noted that, we utilized a more severe incisional pain model that was different from the skin-only incision model. In the model we used, skin plus deep muscle tissues were both simultaneously incised 43. Therefore, the distinct observations of these two studies may attribute to different INC pain models being used.

We still observed some residual pain responses existed in *St2*^-/-^ mice or IL-33 neutralizing antibody-treated mice after skin incision (as shown in Fig. 3A-G). This finding may reflect a complicated inflammatory condition existed in the incised tissues. During tissue injury, a variety of inflammatory mediators, cytokines and chemokines are produced to contribute to the overall pain and inflammation in the inflamed tissues. We found that *Ccl2* expression was significantly up-regulated in the incised skin tissues (Supplementary Fig. 4A). CCL2 is a chemokine that attracts macrophage via CCR2. CCL2 can produce pain by attracting macrophages to the sensory neurons/nerves or by directly activating sensory neurons via CCR2 79, 80. We found blocking CCR2 significantly alleviated INC pain, suggesting an involvement of CCL2/CCR2 signaling in INC pain mechanism (Supplementary Fig. 7A-C). Therefore, there are other potential inflammatory signaling, in addition to IL-33/ST2, involved in mediating INC pain, reflecting the complexity of INC pain mechanisms. However, it is interesting to note that the overexpression of *Ccl2*, as well as some other inflammation-related genes, is significantly reduced in *St2*^-/-^ mice after skin incision. This suggests that IL-33/ST2 signaling can boost inflammation during skin incision, a finding similar with results derived from other types of inflammations 21, 24, 81, 82. Therefore, targeting ST2 signaling may be a better way to ameliorate both pain and inflammation in INC pain.

We found that specifically knocking down *Trpa1* gene expression in neurons innervating incised skin tissues did not significantly affect SOD and GSH levels in the incised skin tissues. This result indicates that ROS level is similar in the incised skin tissues of control and *Trpa1* gene knockdown group. Furthermore, this result also indicates that neuronal TRPA1 does not contribute to oxidative stress in INC model mice. It is known that Schwann cell TRPA1 activation contributes to oxidative stress via NOX1-dependent H_2_O_2_ release from Schwann cells 83. However, silencing TRPA1 in nociceptors only attenuated mechanical allodynia, but without affecting oxidative stress in the inflamed tissues 83. Therefore, our results are consistent with this previous finding, demonstrating that neuronal TRPA1 only contributes to mechanical allodynia but not oxidative stress in INC model mice. Macrophage-derived ROS has been shown to activate TRPA1 expressed in sensory neurons to elicit pain under several chronic pain conditions, including bone cancer pain, complex regional pain syndrome, neuropathic pain and fibromyalgia 79, 84-87. Our study further expanded this notion to INC pain and found that macrophage-derived ROS can activate TRPA1 innervating the incised tissues to contribute to INC pain. Therefore, it is likely that regardless of the initiator, the final common pathway involving macrophages, ROS, and TRPA1 plays an important role in mediating pain underlying a variety of tissue injury or nerve damage conditions.

IL-33 has been reported to contribute to wound healing 88. Application of IL-33 promotes wound healing, whereas *Il33* knockout mice showed delay in wound healing. Mechanistically, IL-33 can promote M2 macrophage polarization and infiltration of innate lymphoid cells, resulting in proliferation of fibroblasts, extracellular matrix deposition and early epithelial repairs 89, 90. Moreover, IL-33 can suppress NF-κB signaling to inhibit excessive inflammation and maintain keratinocyte proliferation 91. It is further reported that the nuclear IL-33, but not IL-33 as a cytokine, exerts beneficial effects on wound healing, since blocking the intracellular signaling of IL-33 does not affect wound healing process in mice 91. Furthermore, we observed that *Il33* expression in incisional skin was not significantly changed in *St2*^-/-^ mice vs. WT mice, indicating blocking ST2 signaling did not affect *Il33* expression per se in INC pain model. We next observed that *St2*^-/-^ mice did not show any deficit in wound healing vs. WT mice after skin incision. Therefore, our study suggests that blocking ST2 may be a potential therapeutic approach for ameliorating INC pain without affecting wound healing process. Our study further identifies that blocking ROS did not significantly affect wound healing in INC pain model mice. But it is known that ROS play a critical role in killing microorganisms, especially during wound healing process. It still remains likely that blocking IL-33/ST2 signaling or the subsequent ROS production may affect the ability of the immune system to kill microorganisms. Thus, further studies will be needed to evaluate whether antimicrobial ability of the immune system is indeed affected by blocking IL-33/ST2 signaling in INC pain model.

## Conclusions

Our study uncovered a previously unrecognized contribution of IL-33/ST2 signaling in mediating mechanical allodynia and gait impairment of a mouse model of INC pain. Targeting IL-33/ST2 signaling may offer a potential therapeutic approach for future INC pain control.

## Supplementary Material

Supplementary figures.
