# Supplementary material for: IL-33/ST2 induces macrophage-dependent ROS production and TRPA1 activation that mediate pain-like responses by skin incision in mice
Source: Theranostics. 2024 Aug 19;14(13):5281–302. doi: 10.7150/thno.97856 (PMC11388077; doi:10.7150/thno.97856)
Supplement: Supplementary file 1 — Supplementary figures. [file thnov14p5281s1.pdf]

Supplementary figure legends

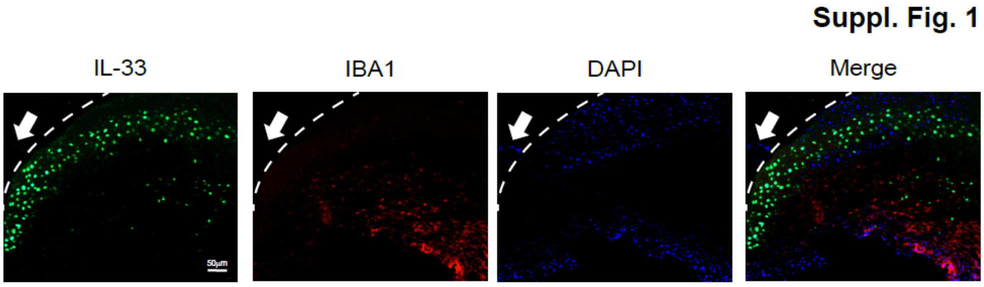

Suppl. Fig. 1 IL-33 was not expressed in skin infiltrating macrophages. Representative immunofluorescence pictures showing IL-33 (in green) and Iba-1 (in red, a macrophage marker) immunostaining in the incised skin of INC pain model mice. DAPI is shown in purple. Scale bar indicates 50 µm. White line denotes stratum corneum of epidermis. White arrows indicate the direction of the incision made.

Suppl. Fig. 2

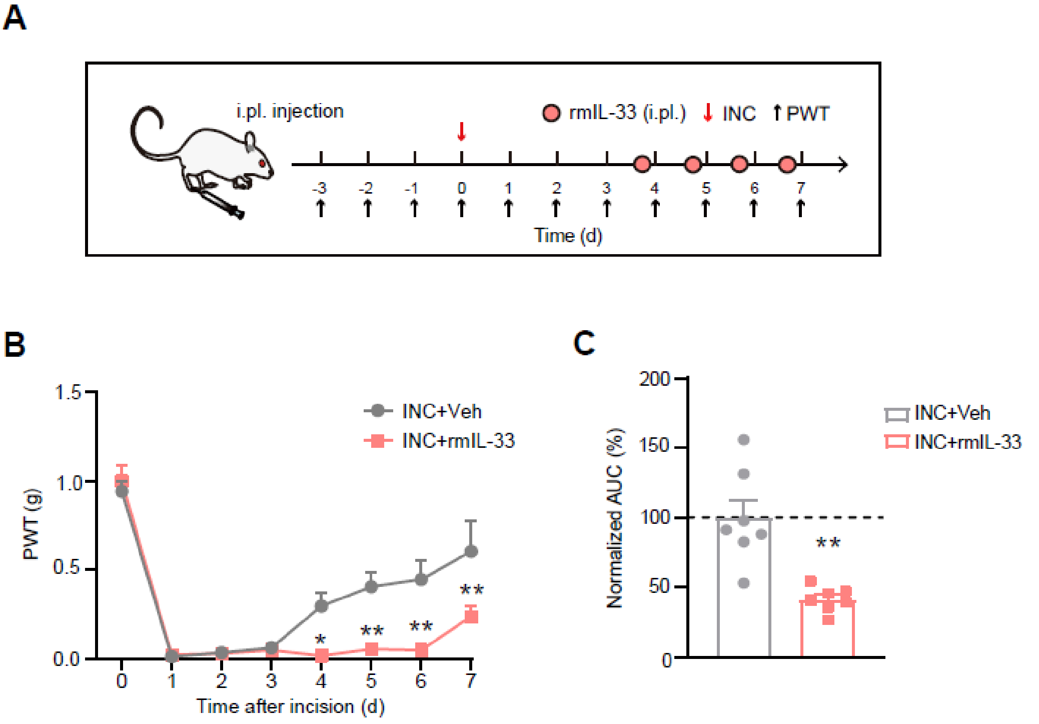

Suppl. Fig. 2 Recombinant mouse IL-33 (rmIL-33) injection into the incised tissues worsened mechanical allodynia of INC model mice. (A) Schematic picture showing time points for INC model establishment, rmIL-33/vehicle application and behavioral assay. (B) Time course showing PWT changes of INC pain model mice receiving either rmIL-33 (300 ng/site) or corresponding vehicle (0.1% BSA in PBS) injection into the incised tissues. (C) Summary of AUC of curves as shown in panel A. $^{*}p<0.05$ and $^{**}p<0.01$ vs. INC+Veh group. n=7 mice/group. Two-way ANOVA followed with Tukey's post-hoc test in panel A. Student's unpaired *t* test for panel B.

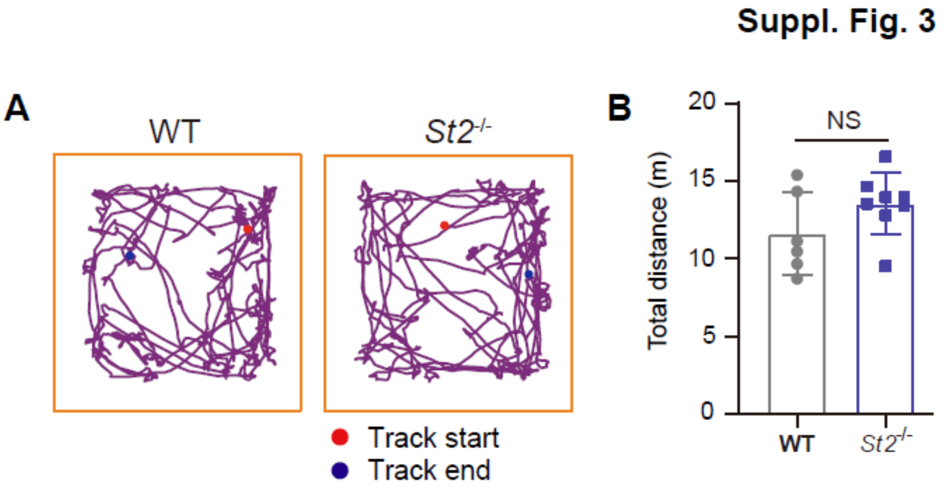

Suppl. Fig. 3 *St2*$^{-/-}$ mice displayed normal locomotor activity. (A) Representative movement traces of WT and *St2*$^{-/-}$ mouse in the open field test. (B) Summary of the total distance travelled by two groups of mice. n=6-8 mice/group. NS: no significance. Student's unpaired *t* test for panel B.

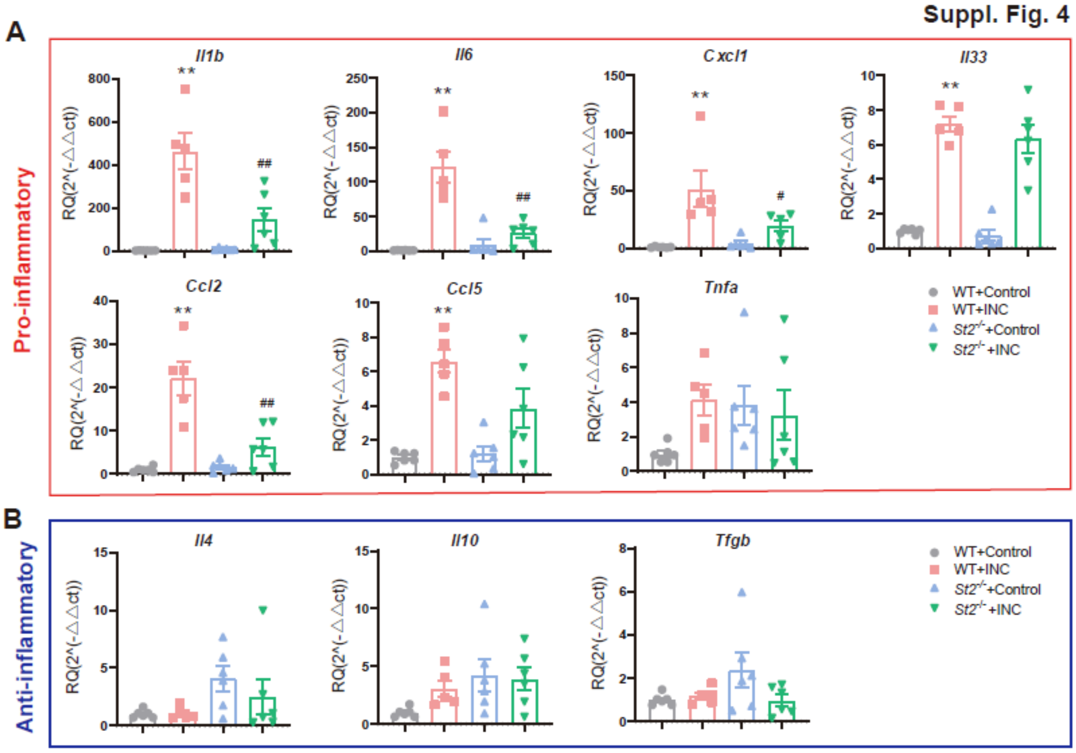

Suppl. Fig. 4 Expression changes of certain pro-inflammatory and anti-inflammatory genes in WT and *St2*$^{-/-}$ mice upon incision. (A) Expression changes of some typical pro-inflammatory genes in skin tissues of WT+Control, WT+INC, *St2*$^{-/-}$+Control and *St2*$^{-/-}$+INC groups of mice. (B) Expression changes of some typical anti-inflammatory genes in skin tissues of WT+Control, WT+INC, *St2*$^{-/-}$+Control and *St2*$^{-/-}$+INC groups of mice. n=5-6 mice/group. $^{**}p<0.01$ vs. WT+Control group. $^{\#}p<0.05$, $^{\#\#}p<0.01$ vs. WT+INC group. One-way ANOVA followed with Tukey's post-hoc test.

Suppl. Fig. 5

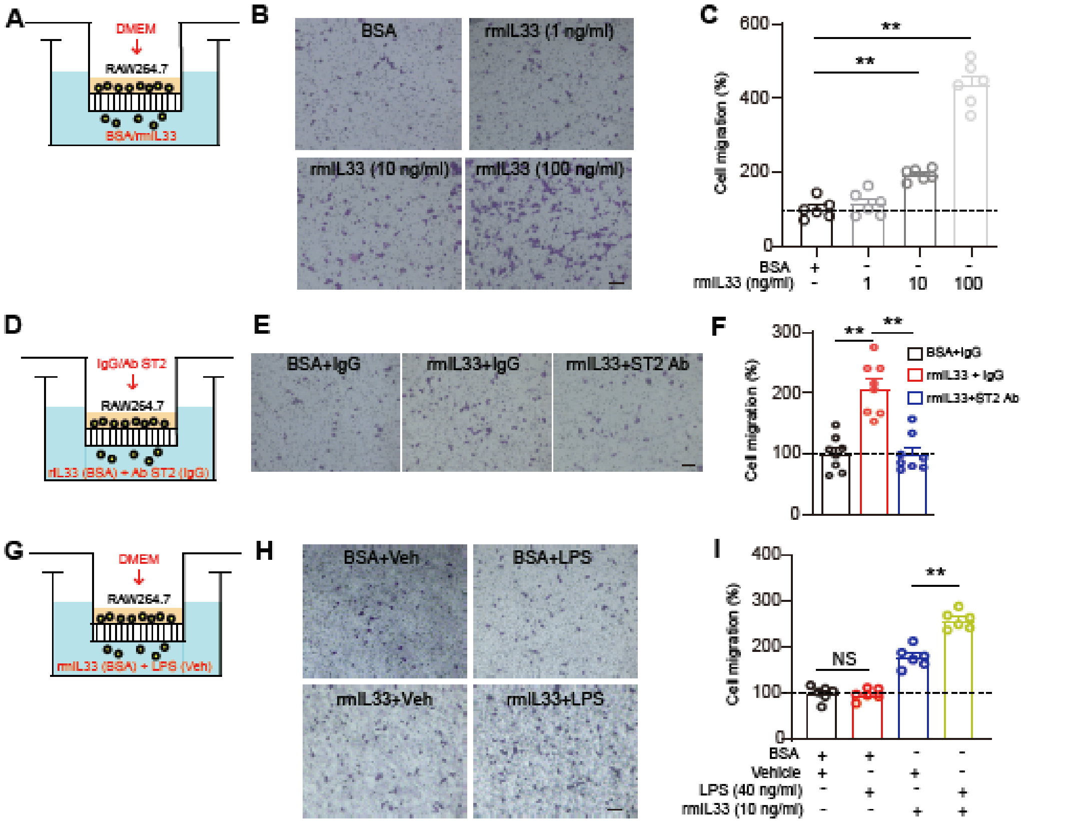

Suppl. Fig. 5 IL-33/ST2 signaling induces macrophage chemoattraction in vitro. (A) Schematic picture showing transwell migration assay using murine macrophage cell line RAW264.7 cell with Boyden chamber. Recombinant IL-33 (rIL-33) or vehicle (BSA) was added in the medium as indicated. (B) Representative photos showing migration of Raw264.7 cells in medium containing BSA or different concentrations of rmIL-33 (1, 10 or 100 ng/ml). (C) Summary of cell migration % as in panel B. Results from BSA-treated group was taken as 100% and all others were normalized. (D) Schematic picture of RAW264.7 cell in Boyden chamber. Recombinant mouse IL-33 (rmIL-33, 10 ng/ml)/BSA and ST2 neutralizing antibody (ST2 Ab, 30 μg/ml)/ isotype control IgG was added in the medium as indicated. (E) Representative photos showing migration of Raw264.7 cells towards medium containing BSA+IgG, rmIL-33+IgG or rmIL-33+ST2 Ab. (F) Summary of cell migration % as in panel E. Results from BSA+IgG group was taken as 100% and all others were normalized. (G) Representative photos showing migration of Raw264.7 cells in medium containing rmIL-33/BSA and LPS/Veh (DMEM). (H) Representative photos showing migration of Raw264.7 cells towards medium containing rmIL-33 (10 ng/ml)/BSA and LPS (40 ng/ml)/Veh. (I) Summary of cell migration % as in panel H. Results from BSA+Veh group was taken as 100% and all other were

normalized. Scale bar=100 µm. n=6-8 tests/group. **p<0.01. NS: no significance. One-way ANOVA followed with Tukey's post-hoc test was used.

Suppl. Fig. 6

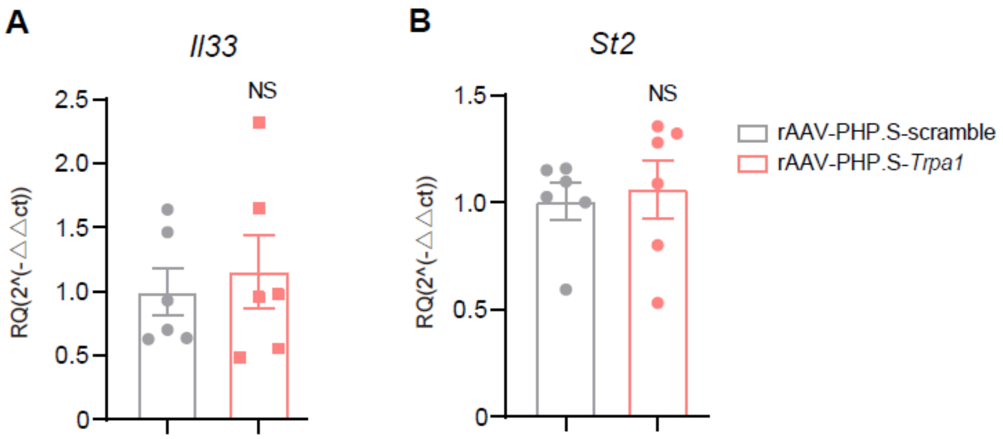

Suppl. Fig. 6 *Il33* and *St2* gene expression in the incised tissues of *Trpa1* knockdown mice. (A&B) qPCR examination of *Il33* and *St2* gene expression in the incised tissues of mice with *Trpa1* knockdown. n=6 mice/group. NS: no significance. Student's unpaired *t* test.

Suppl. Fig. 7

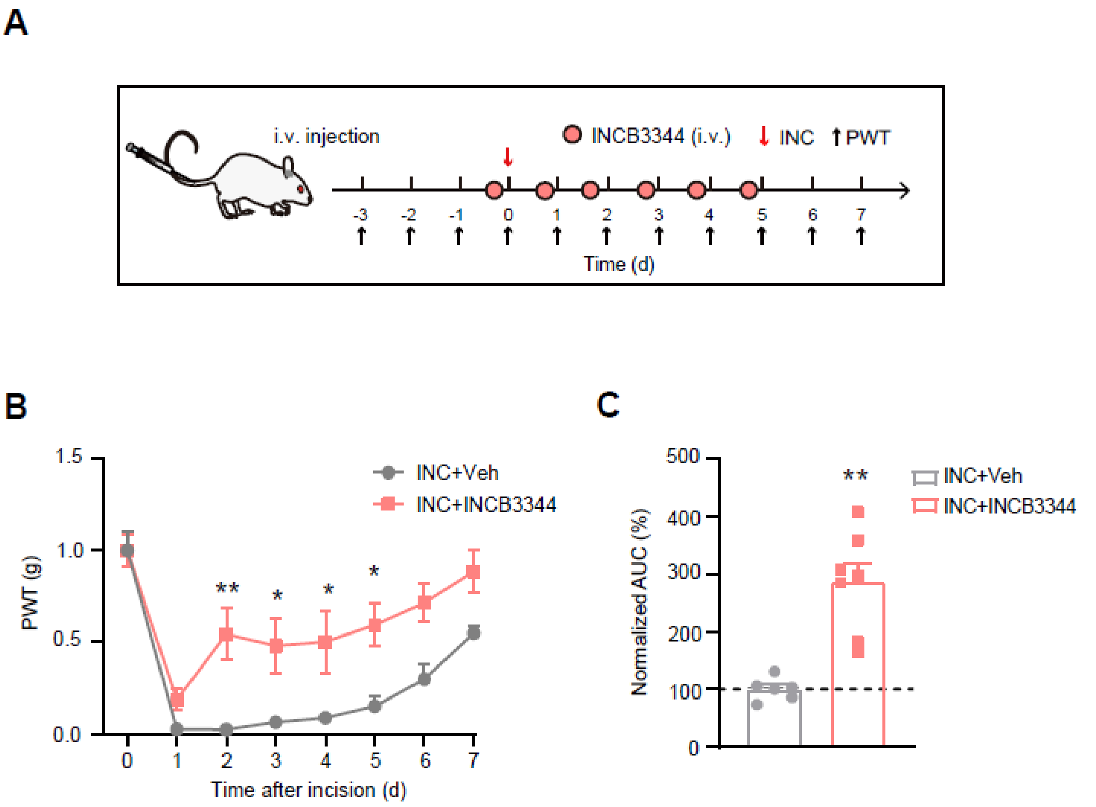

Suppl. Fig. 7 Blocking CCR2 signaling reduces mechanical allodynia in INC pain model mice. (A) Schematic picture showing time points for INC model establishment, INCB3344/vehicle application and behavioral assay. (B) Time course showing PWT changes of INC pain model mice receiving either INCB3344 (a CCR2 specific antagonist) or corresponding vehicle (0.3% DMSO in PBS) treatment (i.v.). (C) Summary of AUC of curves as shown in panel A. $^{*}p<0.05$ and $^{**}p<0.01$ vs. INC+Veh group. n=6-7 mice/group. Two-way ANOVA followed with Tukey's post-hoc test in panel A. Student's unpaired *t* test for panel B.
